# Relationship between personality traits and spontaneous coronary artery dissection risk: evidence from Mendelian randomization

**DOI:** 10.3389/fcvm.2025.1384090

**Published:** 2025-02-12

**Authors:** Kun Zheng, Mengdi Wu, Junhua Wang, Jinjin Sun, Yuqian Li, Peng Wang, Zhiyue Zhang, Xiuming Pan, Yifeng Yang, Tianqi Li, Yujie Guo

**Affiliations:** ^1^ Graduate School, China Medical University, Shenyang, Liao Ning, China; ^2^ Department of Cardiology, Air Force Medical Center, The Fourth Military Medical University, Beijing, China; ^3^ Graduate School, Hebei North University, Zhangjiakou, Hebei, China

**Keywords:** myocardial infarction, spontaneous coronary artery dissection, personality trait, causality, Mendelian randomization

## Abstract

**Background:**

Spontaneous coronary artery dissection (SCAD) significantly contributes to myocardial infarction among young individuals. Despite the elusive nature of its etiology, empirical evidence indicates a substantial correlation between sociopsychological factors and the disorder. This investigation endeavored to discern a genetic basis for personality traits influencing SCAD susceptibility.

**Methods:**

Bidirectional univariate and multivariate Mendelian randomization (MR) analyses were hereby conducted to investigate the putative causal nexus between personality dimensions and SCAD risk. Besides, data regarding SCAD and personality were extracted from expansive genome-wide association studies (GWAS), and rigorous statistical inferences were made using inverse variance weighting (IVW) and ancillary methodologies. Additionally, sensitivity evaluations were performed to bolster statistical assertions.

**Results:**

Univariate MR analyses indicated heightened neuroticism scores as harbingers of increased SCAD risk [Odds Ratio (OR) = 1.31, 95% Confidence Interval (CI): 1.08–1.60, *P* = 0.007], while other personality characteristics revealed no causal interplay with SCAD. After excluding single nucleotide polymorphisms (SNPs) confounded by extrinsic variables, the association of neuroticism scores with SCAD susceptibility persisted. These findings were further substantiated by multivariate MR analyses.

**Conclusions:**

In summary, this study identified a significant association between genetically predicted neuroticism scores and an elevated risk of SCAD. However, additional investigation is still required to elucidate the biological underpinnings of this relationship, as well as the impact of gender, environmental influences, and other contributing factors.

## 1 Introduction

Cardiovascular disease stands out as a leading cause of global mortality, with ischemic heart disease accounting for nearly half of these deaths (1). Spontaneous Coronary Artery Dissection (SCAD) primarily involves the tearing or separation of the coronary artery walls, creating false lumens that compress the true lumen, thereby leading to myocardial ischemia or infarction (2). However, the pathogenesis of SCAD remains unclear, with two prevailing theories: the “inside-out” mechanism and the “outside-in” mechanism (2). The “inside-out” mechanism posits that tearing of the coronary artery's intimal layer allows blood to enter the vessel wall, forming a false lumen that compresses the true lumen and disrupts myocardial perfusion (2, 3). Conversely, the “outside-in” mechanism suggests that spontaneous intramural bleeding leads to the accumulation of blood, and a false lumen that compresses the true lumen is eventually formed (2, 3). SCAD is an increasingly recognized cause of acute coronary syndrome, notably contributing to myocardial infarction in the younger demographic (4, 5). SCAD etiology, involving multiple factors, is rather complex and has not been fully elucidated. Genetic predispositions are evident, with studies highlighting a higher incidence in individuals with a family history of SCAD and identifying various genetic risk loci (6). Additionally, conditions like fibromuscular dysplasia (FMD) and connective tissue disorders, such as Marfan syndrome, contribute to arterial wall fragility, further increasing dissection risks (7). Hormonal factors, especially notable in women during pregnancy and postpartum periods, can trigger SCAD due to hormonal fluctuations (8). Furthermore, psychological and physiological stressors including emotional stress and hemodynamic instability are significant risk factors.

As innately consistent and stable characteristics, personality traits demonstrate enduring predictive value for psychological outcomes including educational attainment and mental health (9). The “Big Five” taxonomy, known as a prevalent model in psychological research, categorizes personality into five dimensions, including conscientiousness, extraversion, openness, neuroticism, and agreeableness (9). Evidence from various studies highlights a robust correlation between personality traits and cardiovascular disease, with the “Big Five” model proving more efficacious in prognosticating health outcomes compared to other typologies (10). Nevertheless, there are notable etiological differences between SCAD and traditional cardiovascular diseases. Research investigating the ties between SCAD and psychosocial factors such as emotional stress and mood disorders yields heterogeneous findings (11, 12). Hence, the nexus between personality traits and SCAD warrants additional scrutiny.

Mendelian randomization (MR) leverages genetic variants associated with exposures to ascertain their effects on outcomes. This robust method circumvents the residual confounding and reverse causality often encountered in observational research, facilitating the exploration of potential causal links between personality traits and SCAD risk (13). In the present study, a two-sample MR analysis was performed to assess the genetic underpinnings of personality traits concerning SCAD. Additionally, the study also endeavored to confirm whether these associations were independent of arterial blood pressure.

## 2 Methods

### 2.1 Data sources

The UK Biobank project represents an unprecedented prospective cohort endeavor, amassing extensive genetic and phenotypic information from around half a million participants aged 40–69 across the UK (14). Herein, neuroticism scores were derived from a genome-wide association analysis (GWAS) by Neale Lab, focusing on phenotypes from the UK Biobank (available at http://www.nealelab.is/uk-biobank). Data regarding extraversion, openness, agreeableness, and conscientiousness were sourced from the Genetics of Personality Consortium, which coordinated vast GWAS endeavors on personality traits (15, 16). Information on hypertension was obtained from FinnGen's extensive cohort study, encompassing over 500,000 individuals (17). The SCAD dataset, considered the most comprehensive GWAS meta-analysis to date, incorporated data from eight studies of European descent. Stringent quality controls were implemented throughout the analytical process (4).

### 2.2 Selection of SNP

In the SNP screening process, a multi-faceted quality control protocol was employed. Initially, instrumental variables strongly linked to the exposures (*P* < 5 × 10^−8^) in each GWAS summary dataset were identified. For the traits of extraversion, openness, agreeableness, and conscientiousness, SNPs at this stringent threshold were limited. Hence, SNPs with *P* < 5 × 10^−5^ were selected, consistent with prior psychiatric MR studies (18, 19). Subsequently, they were clumped utilizing a threshold of r^2^ < 0.001 and a distance greater than 10,000Kb to mitigate linkage disequilibrium. Furthermore, SNPs related to the exposures (*P* < 5 × 10^−8^) were excluded. Reviewing the literature revealed hypertension as a risk factor for SCAD. Consequently, the Pheno Scanner database 2qw used to exclude SNPs associated with confounding factors (http://www.phenoscanner.medschl.cam.ac.uk/phenscanner) (4, 5, 20). Finally, to ensure instrument validity, the F-statistic was employed to assess instrument strength, discarding SNPs with an F-value below 10 (21).

### 2.3 Statistical analyses

In the univariate Mendelian randomization framework (UVMR), the inverse variance weighted (IVW) method was primarily employed for statistical inference. To address potential heterogeneity and pleiotropy, the MR Egger, weighted median, and MR-PRESSO were utilized as complementary analytical approaches (22). MR-PRESSO effectively detected and corrected outliers, refining the analysis, while MR-Radial further supported and strengthened the robustness of MR-PRESSO (23). Besides, horizontal pleiotropy assessment was conducted using the MR Egger intercept and MR-PRESSO Global Test. Consistency of the results was corroborated through heterogeneity evaluations conducted using both the IVW and MR Egger methods (24). Drawing from the established SNP selection protocol, the causal influence of SCAD on five personality dimensions was explored using reverse MR analyses.

Existing observational research has delineated an association between personality traits and hypertension, i.e., an established risk factor for SCAD (4, 25). The present investigation employed multivariate Mendelian randomization (MVMR) to discern the effects of individual personality traits on SCAD, independent of hypertension (26). Following the removal of non-robust instrumental variables, the IVW approach served as the primary method of analysis. Ultimately, the MR Egger, weighted median, MR-PRESSO, and MR-LASSO methods were applied to ensure the integrity of the findings, thereby safeguarding against result variability and detecting any horizontal pleiotropy and heterogeneity (26).

Mendelian randomization analyses were quantified by odds ratios (ORs), 95% confidence intervals (CIs), and *P*-values, with adjustments for multiple testing using Bonferroni correction. The Bonferroni correction, known for its stringency, suggested suggestive of an association at a *P*-value threshold between 0.01 (adjusted for five exposures) and 0.05 (27). Additionally, all statistical analyses were executed using the “TwoSampleMR”, “MendelianRandomization”, “MVMR”, and “RadialMR” packages within R software, version 4.2.3.

## 3 Results

### 3.1 Univariable MR

In this study, UVMR analyses were carried out following the exclusion of outliers detected by MR Radial and MR PRESSO methods. A series of 26–50 SNPs were employed, and no substantial weak instrument bias was noted (F > 10). Details regarding the SNPs utilized and those omitted are provided in Supplementary Table S2.

**Figure 1:**
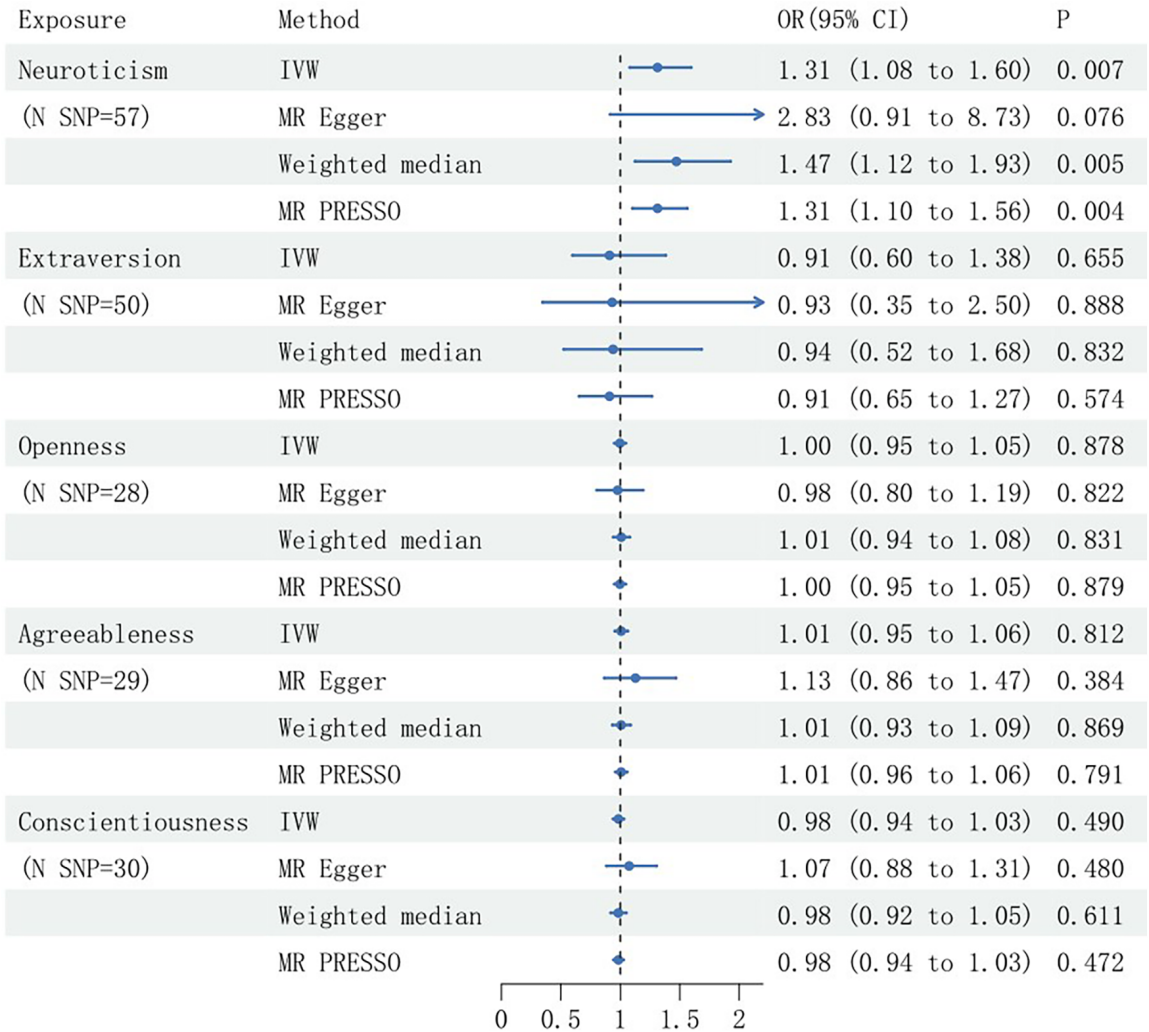
Mendelian randomization estimates of the causal effects of genetically predicted personality traits on spontaneous coronary artery dissection (SCAD), presented with Odds Ratios (OR) and 95% confidence intervals. SNP, single nucleotide polymorphism; N, number; IVW, inverse variance weighted.

IVW analysis indicated a significant association between genetically predicted neuroticism scores and an elevated risk of SCAD, with an OR of 1.31 (95% CI: 1.08–1.60; *P* = 0.007). Similar associations were observed using both the weighted median approach (OR = 1.47; 95% CI: 1.12–1.93; *P* = 0.005) and MR-PRESSO (OR = 1.31; 95% CI: 1.10–1.56; *P* = 0.004). Conversely, MR Egger analysis did not yield significant evidence of an association (OR = 2.83; 95% CI: 0.91–8.73; *P* = 0.076), and no association was observed between genetically determined extraversion or openness and SCAD risk (Figure 1).

**Figure 2:**
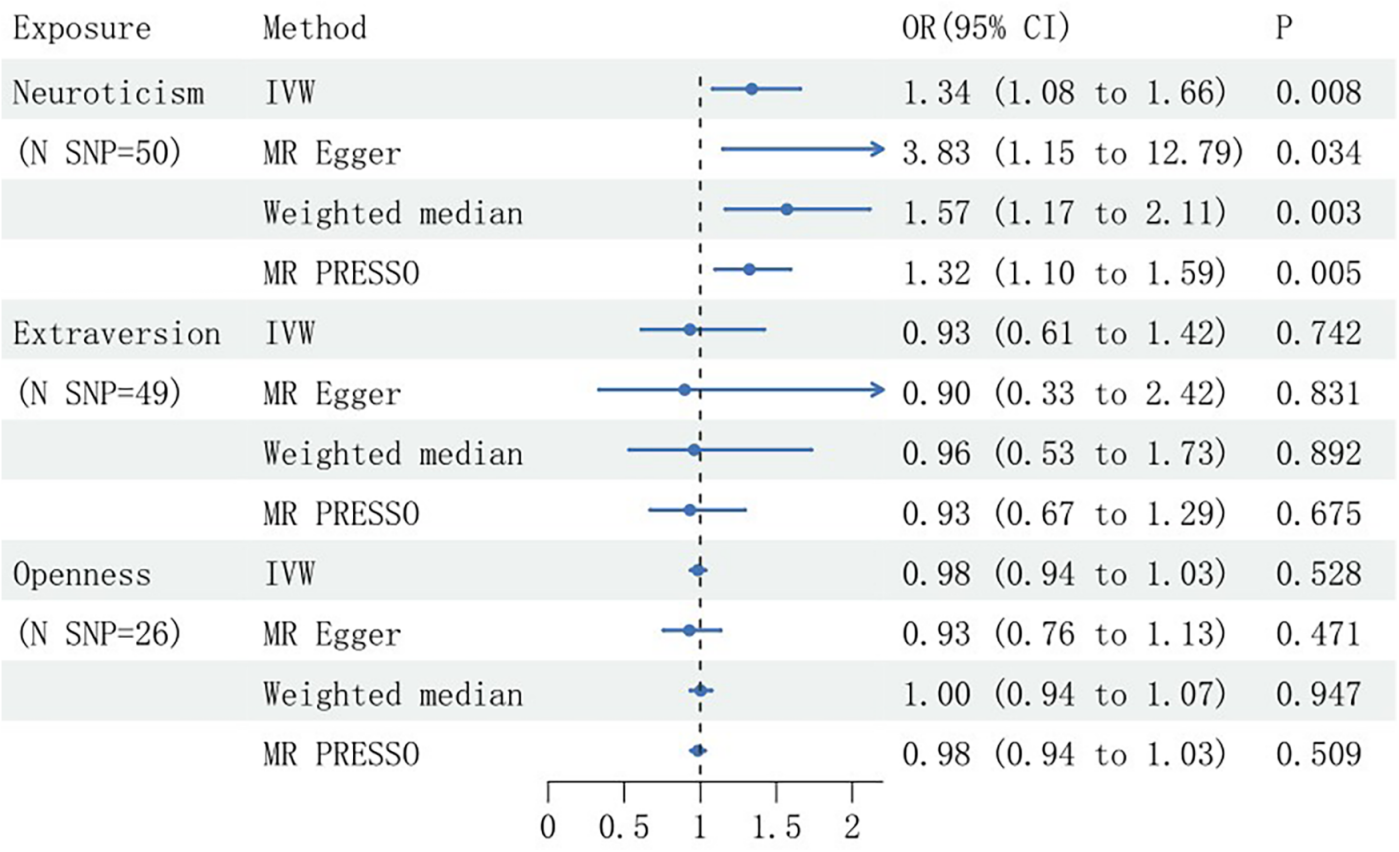
Mendelian randomization estimates of the causal effects of genetically predicted personality traits on spontaneous coronary artery dissection (SCAD), after adjusting for confounders, presented with Odds Ratios (OR) and 95% confidence intervals. SNP, single nucleotide polymorphism; N, number; IVW, inverse variance weighted.

To address potential confounding by hypertension, SNPs associated with hypertension were manually excluded, with subsequent reanalysis conducted. This refined analysis substantiated a significant causal association between genetically proxied neuroticism scores and SCAD (*P* = 0.008), demonstrating a 34% increase in SCAD risk per 1-standard deviation rise in neuroticism. The findings from the weighted median and MR-PRESSO were consistent with the initial results, showing odds ratios (ORs) of 1.57 (95% CI: 1.17–2.11; *P* = 0.003) and 1.32 (95% CI: 1.10–1.59; *P* = 0.005), respectively. Additionally, the MR Egger analysis suggested causally suggestive of an association (OR = 3.83; 95% CI: 1.15–12.79; *P* = 0.034), and no association was observed between genetically determined extraversion or openness and SCAD risk (Figure 2).

This investigation employed a comprehensive sensitivity analysis approach. The MR study revealed no significant heterogeneity or horizontal pleiotropy across genetic variants, as evidenced by *Q*-test, MR-Egger intercept, and MR-PRESSO outcomes. These findings remained unaffected even after adjusting for confounding factors (Table 1). Visual inspections of scatter and residual plots identified no apparent outliers amongst the SNPs representing genetically proxied personality traits. Consequently, the data did not support the presence of horizontal pleiotropy in the genetic associations between personality traits and SCAD.

**Table 1:** Heterogeneity and horizontal pleiotropy of the instrumental variables.

	Exposure	Heterogeneity test		Pleiotropy test	MR-PRESSO	
		Cochran's *Q*-test (*P*-value)		Egger intercept (*P*-value)	Distortion test	Global test
		IVW	MR-Egger	MR-Egger	Outliers	*P*-value
UVMR	Neuroticism	0.85	0.90	0.08	NA	0.85
	Extraversion	0.99	0.98	0.93	NA	0.98
	Openness	0.63	0.59	0.56	NA	0.63
	Agreeableness	0.77	0.76	0.40	NA	0.77
	Conscientiousness	0.62	0.62	0.37	NA	0.63
UVMR (Remove Confounders)	Neuroticism	0.86	0.88	0.18	NA	0.87
	Extraversion	0.98	0.98	0.96	NA	0.98
	Openness	0.48	0.43	0.85	NA	0.48
	Agreeableness	0.77	0.76	0.40	NA	0.77
	Conscientiousness	0.62	0.62	0.37	NA	0.63
MVMR	Neuroticism	0.01	0.01	0.53	NA	0.01

UVMR, univariable MR; MVMR, multivariable MR; IVW, inverse variance weighting.

### 3.2 Multivariable MR

**Figure 3:**
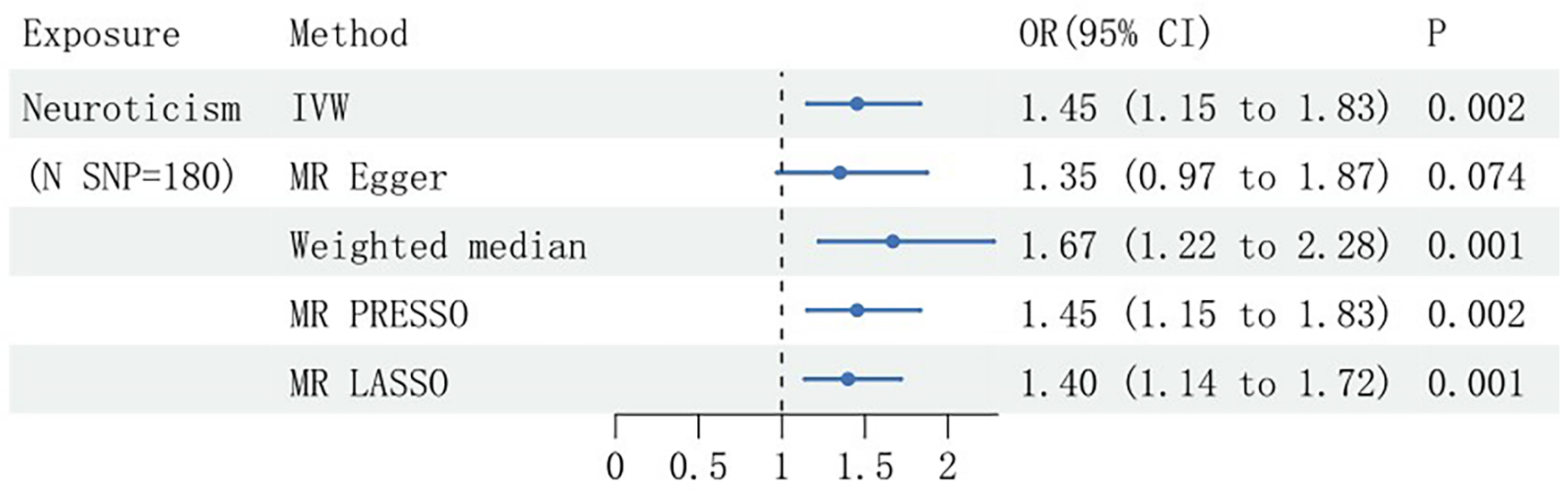
Multivariable Mendelian randomization estimates of the causal effects of neuroticism on spontaneous coronary artery dissection (SCAD), presented with Odds Ratios (OR) and 95% confidence intervals. SNP, single nucleotide polymorphism; N, number; IVW, inverse variance weighted.

In the MVMR, extraversion, openness, agreeableness, and conscientiousness were excluded due to weak instrumental variable strength (F-statistic <10). IVW analysis indicated a robust causal effect of genetically determined neuroticism on SCAD, involving an OR of 1.45 (95% CI: 1.15–1.83, *P* = 0.002), even after adjustment for hypertension. This association was corroborated by results from the weighted median, MR-PRESSO, and MR-LASSO methods, while being not substantiated by MR Egger analysis (Figure 3). Examination for horizontal pleiotropy using MR Egger intercept and MR-PRESSO yielded no substantial findings, but a significant *Q*-test suggested heterogeneity (*P* < 0.05) (Table 1).

### 3.3 Reverse MR

The Reverse MR analysis involved SNPs with counts ranging from 9 to 22 per outcome, all demonstrating robust instrument strength (F-statistic >10) (Supplementary Table S2). The investigation revealed no statistically significant associations between personality traits and ischemic stroke, independent of adjustment for potential confounders (Supplementary Figure S9). Besides, sensitivity analyses provided no evidence of significant heterogeneity or horizontal pleiotropy (Supplementary Table S3).

## 4 Discussion

In this study, MR was employed to investigate the genetic determinants of five personality traits and their causal relationship with SCAD. Univariate MR analyses suggested that elevated genetic predisposition to neuroticism might be associated with an increased risk of SCAD. This association was substantiated in multivariate MR after accounting for potential confounders. Conversely, reverse MR indicated no causal correlation between SCAD and the personality traits under consideration.

Contemporary literature has primarily concentrated on the nexus between personality traits and prevalent cardiovascular conditions. A cornerstone investigation utilizing the UK Biobank cohort revealed that individuals with conscientious and extroverted personality profiles exhibited a reduced likelihood of experiencing myocardial infarction (12). Conversely, a predisposition to neuroticism was found to be associated with an elevated risk of the same condition (12). Complementing these findings, Rukh's research posited that heightened neuroticism, particularly when coupled with depressive symptoms, significantly increased the susceptibility to heart failure and myocardial infarction (10). Indeed, SCAD represents a relatively underexplored area, with its etiology intertwined with psychological elements. The present study contributed to advancing understanding within this domain.

In this investigation, an association between elevated neuroticism scores and an augmented risk of SCAD was observed. As a trait characterized by heightened negative affectivity, including anxiety, fear, irritability, anger, and sadness, neuroticism was notably prevalent among SCAD patients exhibiting various intensities of such emotions, predominantly females (28, 29). As claimed by Murphy et al., individuals with SCAD often experience heightened levels of pain and anxiety, and to a lesser extent, depression, compared to those without the condition (11). Complementary evidence indicates that emotional distress, particularly anxiety, is frequently reported by SCAD patients preceding the event (30). This affective state is hypothesized to correlate with increased catecholamine release, which, in turn, may escalate arterial shear stress, thereby contributing to intimal or vascular rupture (31). Additionally, catecholamine surges can adversely influence myocardial contractility and heighten the propensity for vascular spasm (32). This neuro-emotional pathway potentially elucidates the findings presented herein. Moreover, individuals with neurotic personalities exhibit greater psychological vulnerability, characterized by unpredictability and a diminished sense of control over life events, as well as an increased propensity to anticipate negative outcomes (28). This perceived lack of control may influence various physiological responses. A comprehensive meta-analysis, encompassing over 100 independent studies involving a total of 8,251 participants, demonstrated an association between uncontrollable and unpredictable stressors and higher, less variable levels of daily cortisol output compared to stressors perceived by individuals as more controllable or predictable (33). Numerous reports have documented the adverse vascular effects of adrenal cortex hormones, and the association between SCAD and adrenal cortex hormones has also been described in some case reports (34). In addition, the reduced predictability and controllability of stress events also exacerbate the subsequent impact of negative emotions, increasing the body's response to emotions (28).

Meanwhile, it is noteworthy that biological gender significantly influences personality traits and SCAD. Studies have indicated that females generally score higher on traits linked to neuroticism and agreeableness across most countries. No notable gender differences are observed in openness and conscientiousness, while differences in extraversion are minimal (35, 36). Regarding neuroticism scores, influenced by cultural background, the variance between males and females ranges from small to moderate (37). SCAD predominantly affects young to middle-aged females, with males representing only 10.5% of cases according to recent studies (38, 39). The influence of gender distribution on the link between various personality traits and SCAD complicates causal interpretations.

Despite its contributions, the present study is still subject to certain limitations. First, the absence of individual-level data precluded a thorough investigation into potential non-linear relationships or stratification effects. Second, the GWAS data employed herein were exclusively derived from individuals of European descent, restricting the extrapolation of the results to diverse ethno-racial populations. Third, the current dataset correlating SCAD with personality traits was comparatively limited, constraining the opportunity for multi-database analyses. Fourth, considering that both the exposure and outcome datasets originated from European cohorts, potential sample overlap could introduce bias. However, assessment of this overlap could be rather challenging. Lastly, while personality traits have been extensively recognized for their stability and heritability, numerous studies underscore the consequential role of environmental factors, which may influence the implications of the present findings to a certain degree (28).

## 5 Conclusion

In summary, this study identified a significant association between genetically predicted neuroticism scores and an elevated risk of SCAD. Further investigation should still be conducted to elucidate the biological underpinnings of this relationship, as well as the impact of gender, environmental influences, and other contributing factors.

## Data Availability

The original contributions presented in the study are included in the article/Supplementary Material, further inquiries can be directed to the corresponding author.
