# Supplementary material for: Relationship between personality traits and spontaneous coronary artery dissection risk: evidence from Mendelian randomization
Source: Front Cardiovasc Med. 2025 Feb 12;12:1384090. doi: 10.3389/fcvm.2025.1384090 (PMC11860944; doi:10.3389/fcvm.2025.1384090)
Supplement: Supplementary file 1 [file Datasheet1.pdf]

Supplementary Material

Supplementary Figures and Tables

1 Supplementary Figures

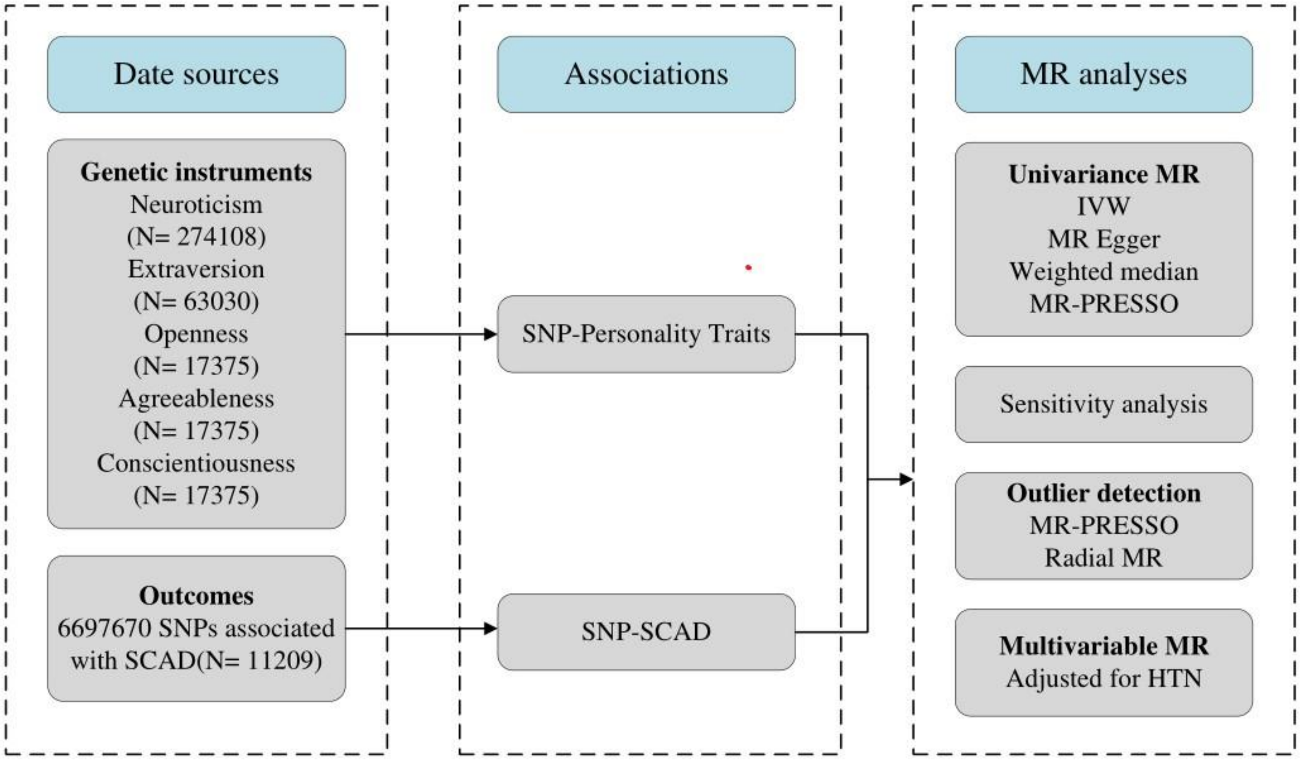

Supplementary Figure 1. Study design. SNPs, single nucleotide polymorphisms; N, number; SCAD, Spontaneous coronary artery dissection; HTN, hypertension; IVW: inverse variance weighted.

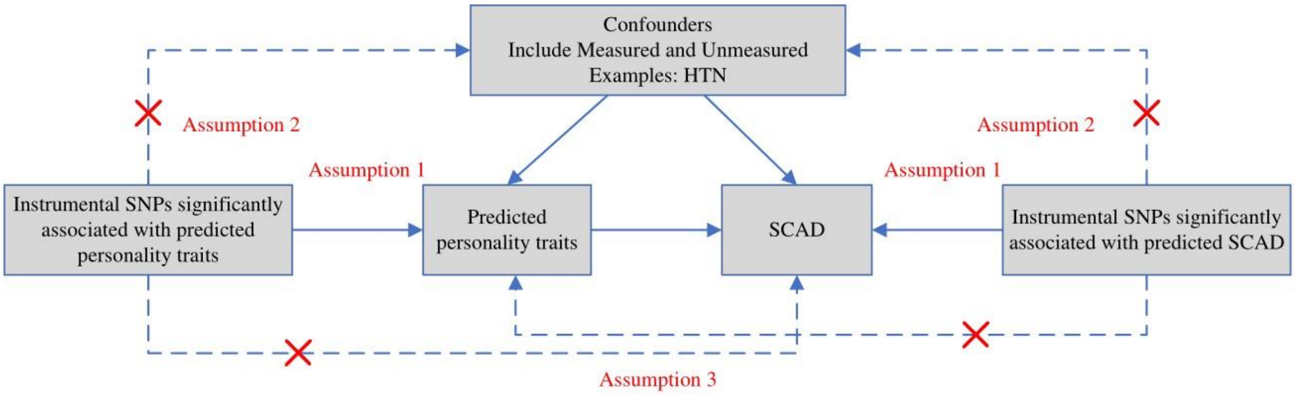

Supplementary Figure 2. Schematic diagram showing the assumptions of Mendelian randomization analysis. SNPs, single nucleotide polymorphisms; SCAD, Spontaneous coronary artery dissection; HTN, hypertension. Assumption 1, instrumental SNPs are robustly associated with the exposure.

Assumption 2, instrumental SNPs are not associated with any potential confounders. Assumption 3, instrumental SNPs cannot directly affect the outcome except through the exposure.

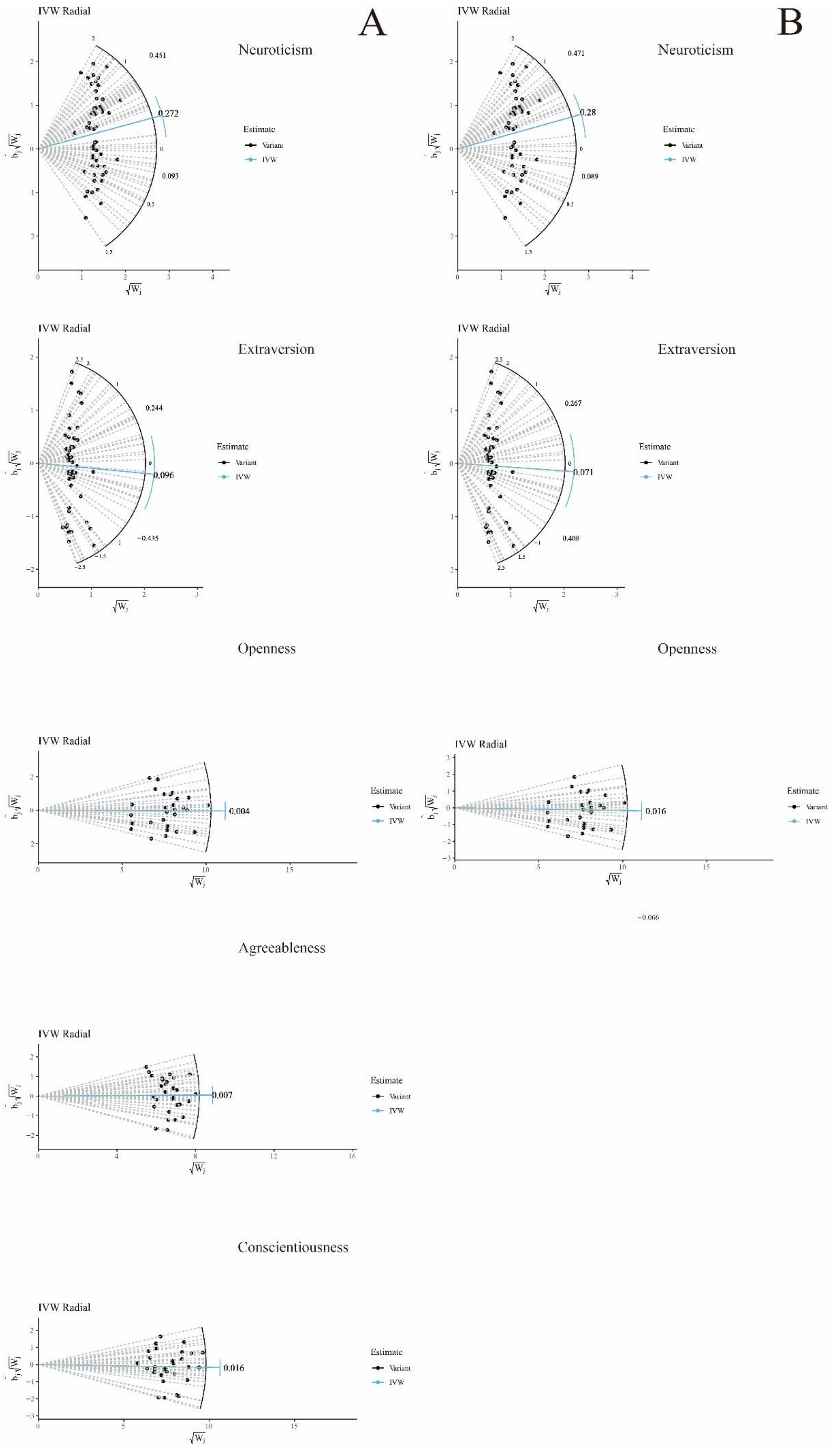
A
B
Neuroticism
IVW Radial
0.451
0.272
0.093
0.471
0.28
0.089
Extraversion
0.244
0.096
−0.435
0.267
0.071
0.408
Openness
0.004
0.016
−0.066
Agreeableness
0.007
Conscientiousness
0.016
Estimate
Variant
IVW

Supplementary Figure 3. Radial plot of the impact of personality traits on SCAD. Without removing confounding factors(A); Confounding factors have been removed(B). SCAD, Spontaneous coronary artery dissection; IVW: inverse variance weighted.

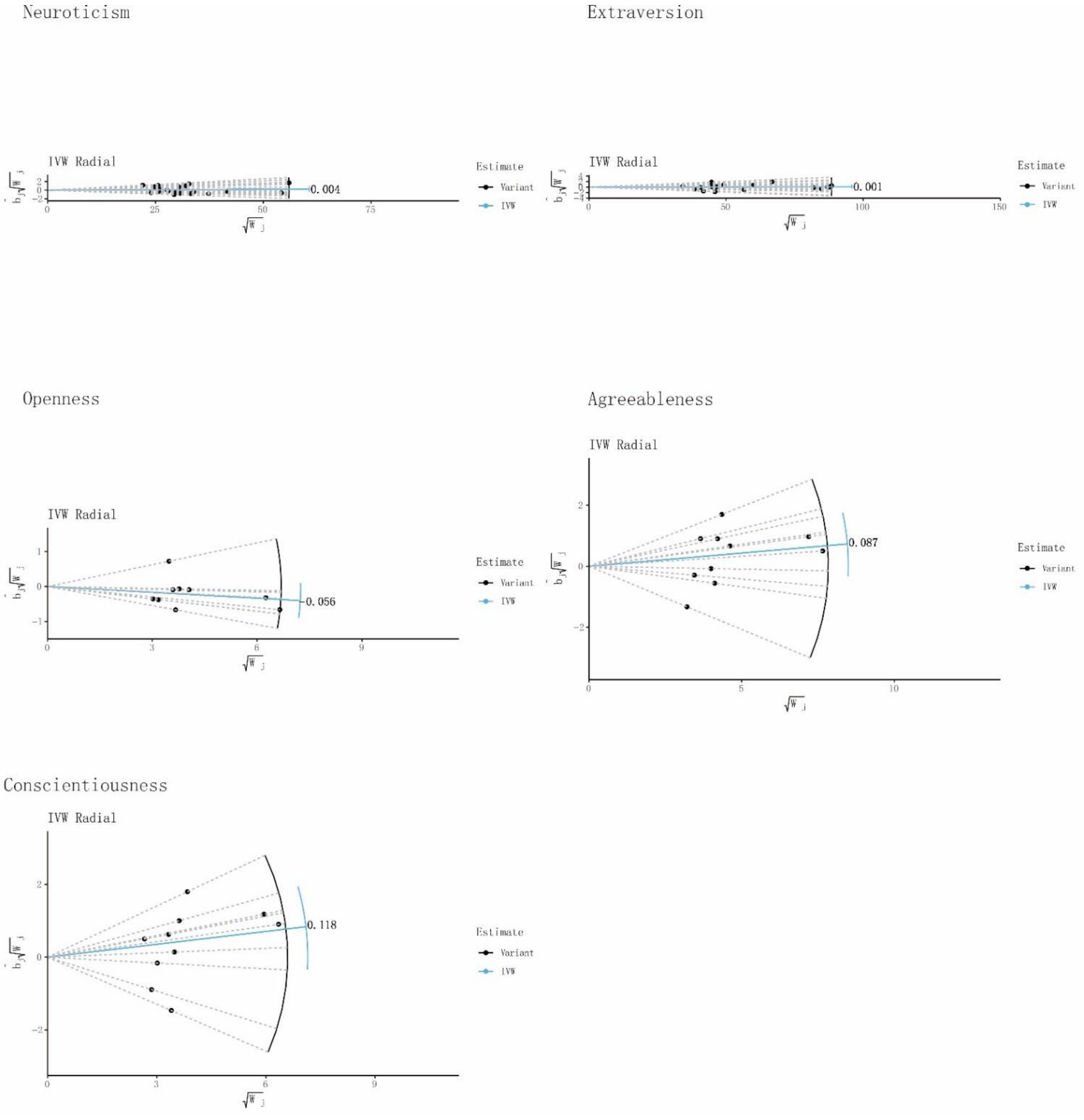

Supplementary Figure 4. Radial plot of the impact of SCAD on personality traits. Without removing confounding factors(A); Confounding factors have been removed(B). SCAD, Spontaneous coronary artery dissection; IVW: inverse variance weighted.

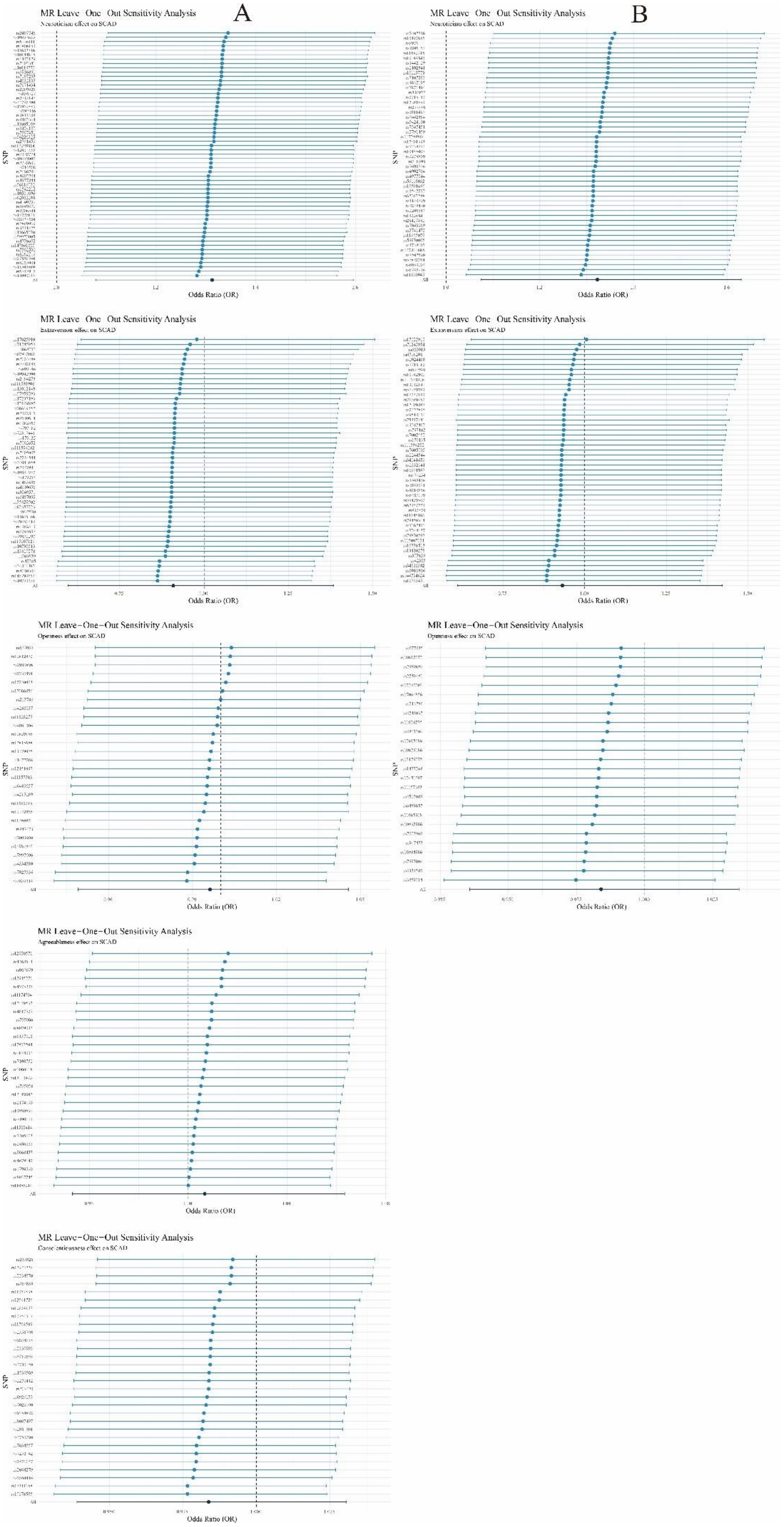
MR Leave One Out Sensitivity Analysis
A
Neuroticism effect on SCAD
MR Leave One Out Sensitivity Analysis
B
Neuroticism effect on SCAD
MR Leave One Out Sensitivity Analysis
Extraversion effect on SCAD
MR Leave One Out Sensitivity Analysis
Extraversion effect on SCAD
MR Leave−One−Out Sensitivity Analysis
Openness effect on SCAD
MR Leave−One−Out Sensitivity Analysis
Openness effect on SCAD
MR Leave−One−Out Sensitivity Analysis
Agreeableness effect on SCAD
MR Leave−One−Out Sensitivity Analysis
Conscientiousness effect on SCAD
SNP
Odds Ratio (OR)

Supplementary Figure 5. Mendelian randomization Leave-One-Out Sensitivity Analysis of the impact of SCAD on personality traits. Without removing confounding factors(A); Confounding

factors have been removed(B). SCAD, Spontaneous coronary artery dissection; MR, Mendelian randomization.

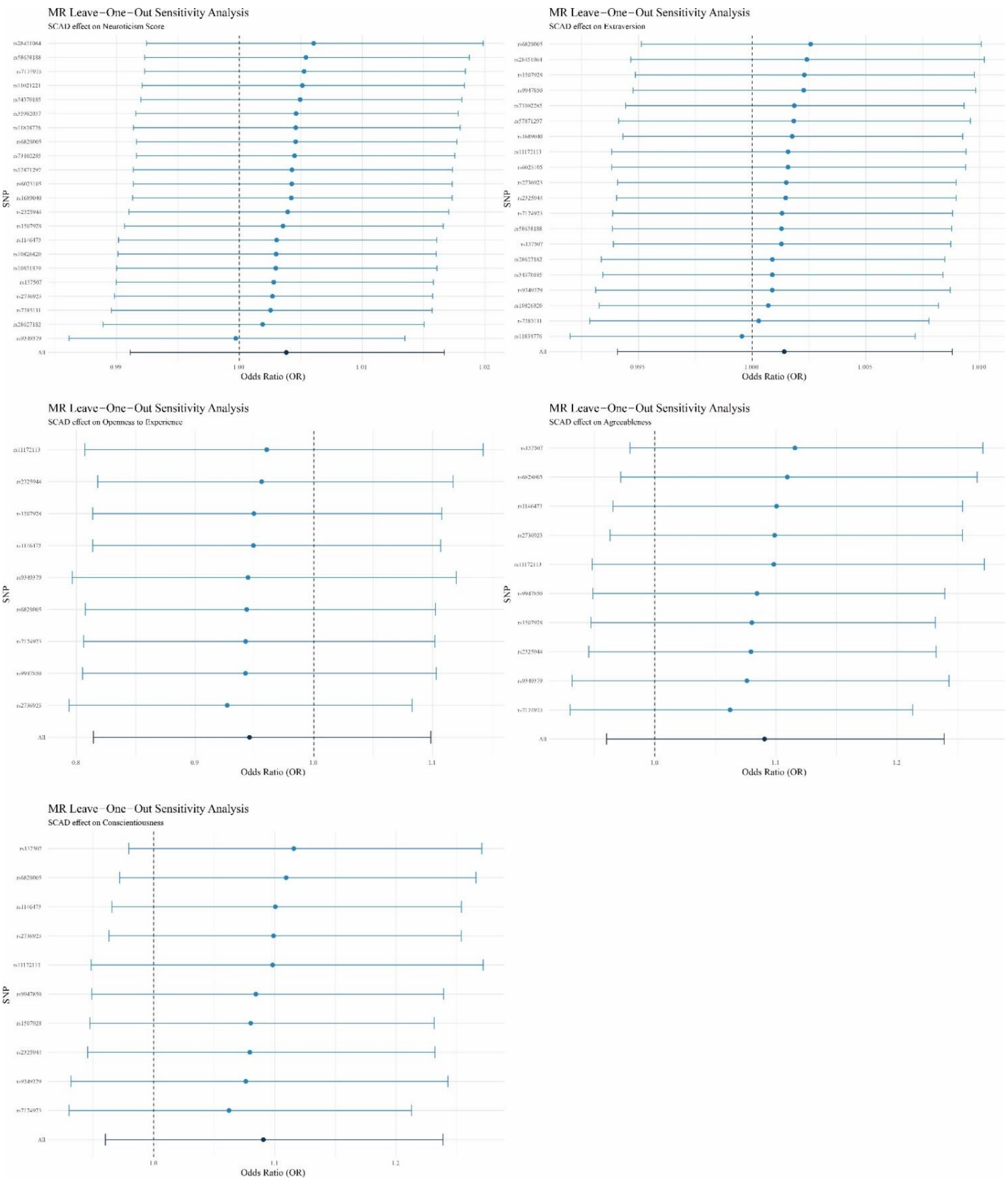

Supplementary Figure 6. Mendelian randomization Leave-One out Sensitivity Analysis of the impact of personality traits on SCAD. SCAD, Spontaneous coronary artery dissection; MR, Mendelian randomization.

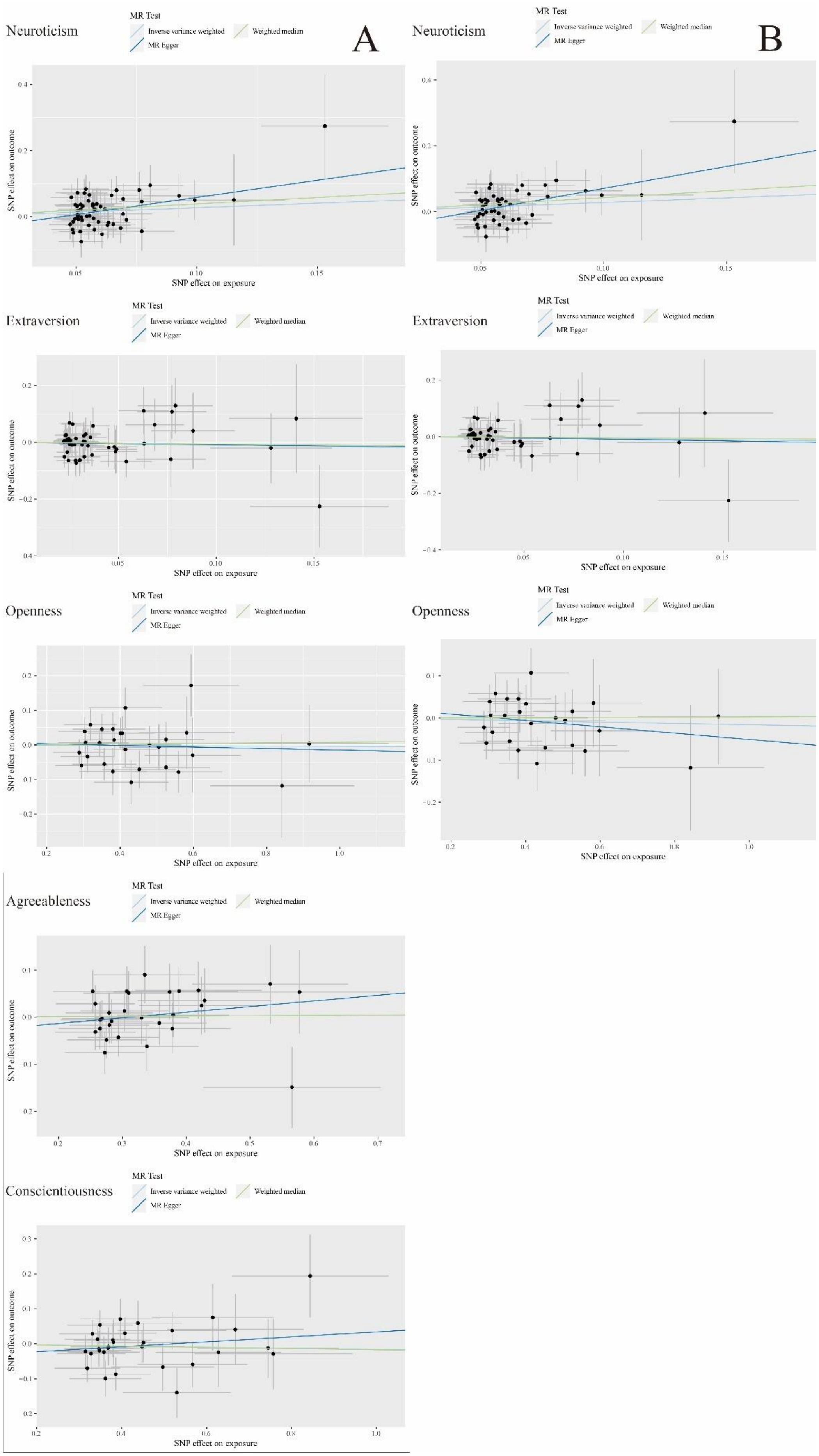
A
B
Neuroticism
Extraversion
Openness
Agreeableness
Conscientiousness
MR Test
Inverse variance weighted
Weighted median
MR Egger
SNP effect on outcome
SNP effect on exposure

Supplementary Figure 7. Scatter plot of the impact of SCAD on personality traits. Without removing confounding factors(A); Confounding factors have been removed(B). SCAD, Spontaneous coronary artery dissection; MR, Mendelian randomization.

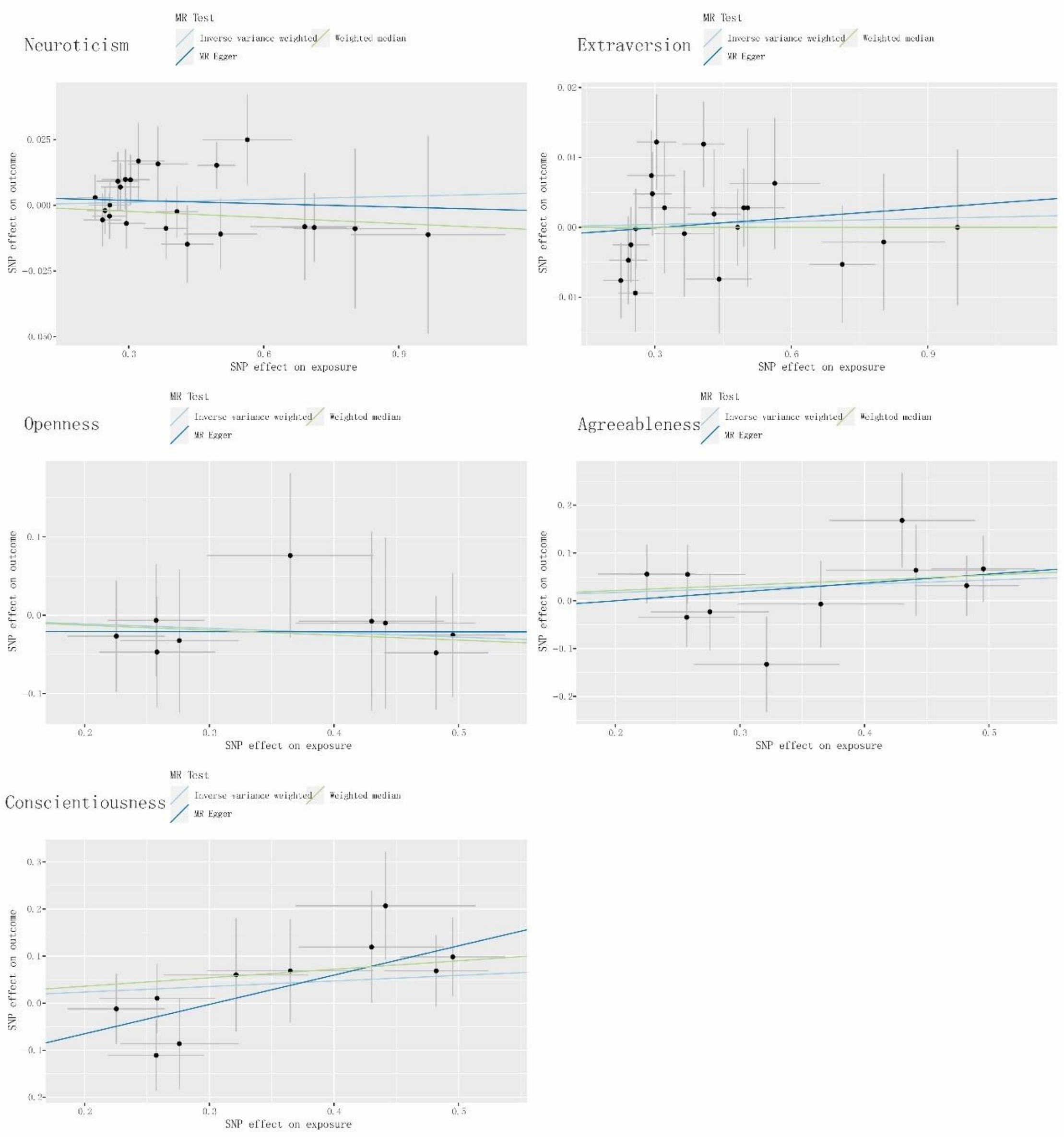

Supplementary Figure 8. Scatter plot of the impact of personality traits on SCAD. SCAD, Spontaneous coronary artery dissection; MR, Mendelian randomization.

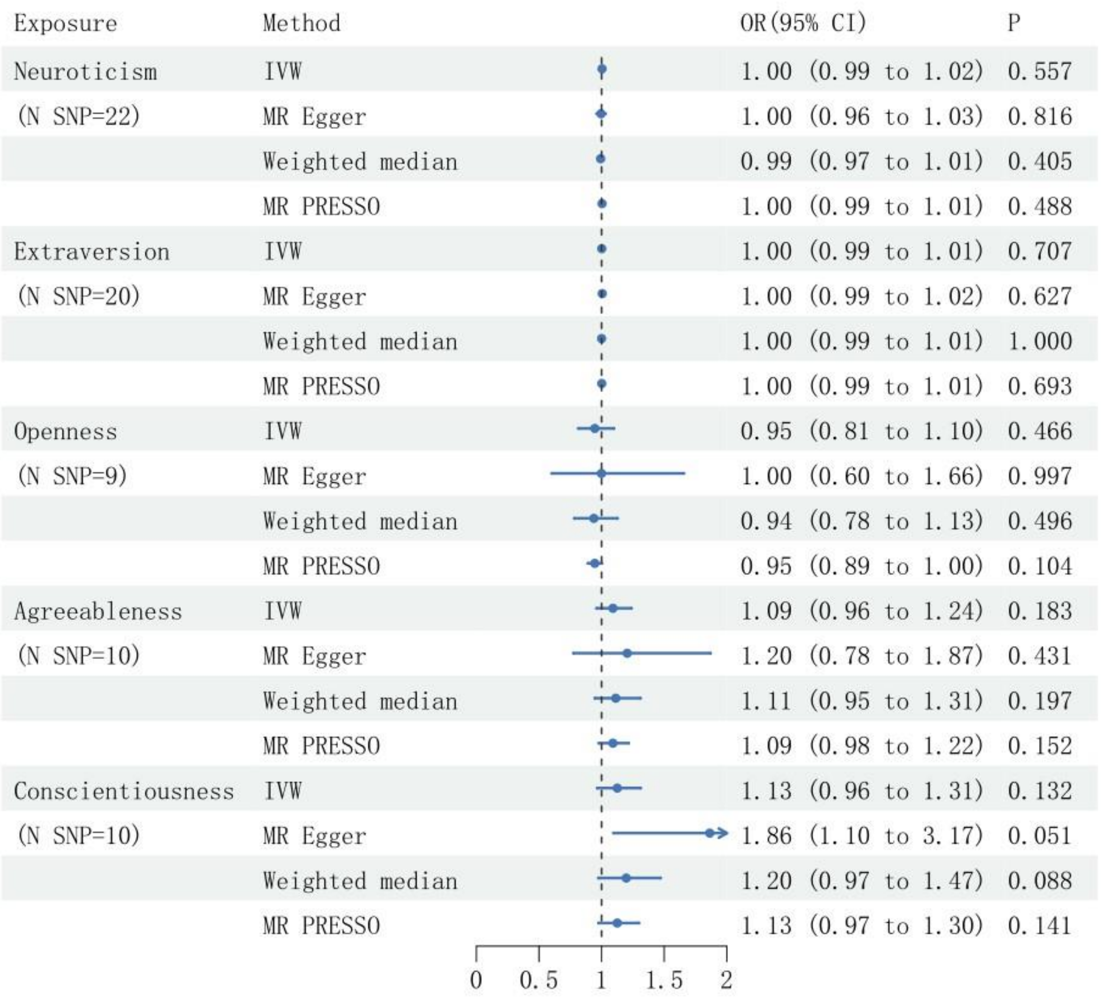

Supplementary Figure 9. Mendelian randomization estimates of the causal effects of personality traits on spontaneous coronary artery dissection (SCAD), presented with Odds Ratios (OR) and 95% confidence intervals. SNP, single nucleotide polymorphism; N, number; IVW: inverse variance weighted.

2 Supplementary Tables

Supplementary Table1. Data sources for the summary statistics.

**Phenotype**	**Sample N**	**SNPs N**	**Ancestry**	**Association model**	**Data Source**
Neuroticism	274,108	10,894,596	European	Linear	UK Biobank
Extraversion	63,030	6,941,603	European	Linear	Genetics of Personality Consortium

Openness	17,375	2,305,641	European	Linear	Genetics of Personality Consortium
Agreeableness	17,375	2,305,462	European	Linear	Genetics of Personality Consortium
conscientiousness	17,375	2,305,683	European	Linear	Genetics of Personality Consortium
Hypertension	377,277	20,170,234	European	Linear	FinnGen study
Spontaneous coronary artery dissection	11,209	6,697,670	European	Logistic	multiple studies including DISCO-3C, SCAD-UK I, SCAD-UK II, Mayo Clinic, DEFINE-SCAD, CanSCAD/MGI, VCCRI I and VCCRI II

N, number.

Supplementary Table2. Summary Table of single nucleotide polymorphisms(SNPs) Used in Statistical Analysis.

	Exposure	Outcome	SNP	A1	A2	BETA	SE	P	R2	F	Radial	Confounder
1	Neuroticism	SCAD	rs10032297	T	A	5.50E-02	8.92E-03	7.11E-10	1.42E-05	3.80E+01	FALSE	TRUE
2	Neuroticism	SCAD	rs10119773	G	A	5.17E-02	8.81E-03	4.39E-09	1.29E-05	3.44E+01	TRUE	TRUE
3	Neuroticism	SCAD	rs10144845	T	C	6.53E-02	9.33E-03	2.49E-12	1.84E-05	4.91E+01	TRUE	TRUE
4	Neuroticism	SCAD	rs10455007	C	A	-5.75E-02	9.01E-03	1.79E-10	1.52E-05	4.07E+01	TRUE	TRUE
5	Neuroticism	SCAD	rs10497655	C	T	-5.21E-02	9.30E-03	2.21E-08	1.17E-05	3.13E+01	TRUE	TRUE
6	Neuroticism	SCAD	rs10501696	G	A	-5.04E-02	8.93E-03	1.67E-08	1.19E-05	3.18E+01	TRUE	TRUE
7	Neuroticism	SCAD	rs10896636	G	C	6.13E-02	9.18E-03	2.42E-11	1.67E-05	4.46E+01	FALSE	FALSE
8	Neuroticism	SCAD	rs11090045	A	G	6.67E-02	9.53E-03	2.61E-12	1.83E-05	4.90E+01	TRUE	TRUE
9	Neuroticism	SCAD	rs11509880	A	G	5.54E-02	9.24E-03	2.00E-09	1.35E-05	3.60E+01	TRUE	FALSE
10	Neuroticism	SCAD	rs11665070	A	G	-7.72E-02	9.26E-03	8.13E-17	2.60E-05	6.94E+01	TRUE	TRUE
11	Neuroticism	SCAD	rs11682716	G	T	-4.84E-02	8.82E-03	4.05E-08	1.13E-05	3.01E+01	TRUE	TRUE

12	Neuroticism	SCAD	rs117298864	A	G	1.15E-01	2.11E-02	4.80E-08	1.12E-05	2.98E+01	TRUE	TRUE
13	Neuroticism	SCAD	rs11805169	A	T	-5.06E-02	9.00E-03	1.81E-08	1.19E-05	3.17E+01	TRUE	FALSE
14	Neuroticism	SCAD	rs12601333	G	C	-5.08E-02	9.06E-03	2.05E-08	1.18E-05	3.15E+01	TRUE	TRUE
15	Neuroticism	SCAD	rs12969553	T	C	-5.72E-02	8.93E-03	1.53E-10	1.54E-05	4.10E+01	TRUE	TRUE
16	Neuroticism	SCAD	rs13226841	C	T	5.72E-02	8.69E-03	4.68E-11	1.62E-05	4.33E+01	TRUE	TRUE
17	Neuroticism	SCAD	rs1442129	G	A	4.88E-02	8.77E-03	2.58E-08	1.16E-05	3.10E+01	TRUE	TRUE
18	Neuroticism	SCAD	rs147861665	A	C	1.53E-01	2.63E-02	5.79E-09	1.27E-05	3.39E+01	TRUE	TRUE
19	Neuroticism	SCAD	rs1542212	G	T	5.68E-02	8.94E-03	2.05E-10	1.51E-05	4.04E+01	TRUE	TRUE
20	Neuroticism	SCAD	rs1673931	C	T	4.95E-02	8.99E-03	3.60E-08	1.14E-05	3.04E+01	FALSE	TRUE
21	Neuroticism	SCAD	rs1806153	T	G	6.84E-02	1.03E-02	3.71E-11	1.64E-05	4.38E+01	TRUE	TRUE
22	Neuroticism	SCAD	rs2102341	C	T	-6.30E-02	9.59E-03	5.23E-11	1.61E-05	4.31E+01	TRUE	TRUE
23	Neuroticism	SCAD	rs2206544	C	T	-5.18E-02	8.78E-03	3.68E-09	1.30E-05	3.48E+01	TRUE	TRUE
24	Neuroticism	SCAD	rs2269426	A	G	6.34E-02	9.05E-03	2.57E-12	1.84E-05	4.90E+01	TRUE	FALSE
25	Neuroticism	SCAD	rs2278609	C	T	6.19E-02	1.06E-02	4.84E-09	1.28E-05	3.43E+01	TRUE	TRUE
26	Neuroticism	SCAD	rs2407746	G	C	6.07E-02	9.47E-03	1.47E-10	1.54E-05	4.11E+01	TRUE	TRUE
27	Neuroticism	SCAD	rs2715147	T	C	4.75E-02	8.69E-03	4.59E-08	1.12E-05	2.99E+01	TRUE	TRUE
28	Neuroticism	SCAD	rs2791459	A	C	-5.06E-02	8.84E-03	1.02E-08	1.23E-05	3.28E+01	TRUE	TRUE
29	Neuroticism	SCAD	rs28427480	C	A	9.26E-02	1.49E-02	5.14E-10	1.45E-05	3.86E+01	TRUE	TRUE
30	Neuroticism	SCAD	rs2921036	C	T	-8.43E-02	8.73E-03	4.86E-22	3.49E-05	9.32E+01	FALSE	FALSE
31	Neuroticism	SCAD	rs297346	G	A	-5.08E-02	9.08E-03	2.15E-08	1.17E-05	3.14E+01	TRUE	TRUE
32	Neuroticism	SCAD	rs34796300	C	T	-5.25E-02	8.79E-03	2.39E-09	1.33E-05	3.56E+01	TRUE	FALSE
33	Neuroticism	SCAD	rs3741475	A	G	6.95E-02	1.10E-02	2.55E-10	1.50E-05	4.00E+01	TRUE	TRUE
34	Neuroticism	SCAD	rs3811489	C	T	4.95E-02	8.87E-03	2.43E-08	1.17E-05	3.11E+01	TRUE	TRUE
35	Neuroticism	SCAD	rs3849470	C	T	-4.96E-02	8.70E-03	1.17E-08	1.22E-05	3.25E+01	TRUE	TRUE
36	Neuroticism	SCAD	rs4140799	A	G	-5.68E-02	8.72E-03	7.53E-11	1.59E-05	4.24E+01	TRUE	TRUE

37	Neuroticism	SCAD	rs4632195	T	C	5.92E-02	8.71E-03	1.09E-11	1.73E-05	4.62E+01	TRUE	TRUE
38	Neuroticism	SCAD	rs4738602	A	G	4.80E-02	8.74E-03	4.03E-08	1.13E-05	3.01E+01	TRUE	TRUE
39	Neuroticism	SCAD	rs4902704	C	G	5.04E-02	8.92E-03	1.63E-08	1.20E-05	3.19E+01	TRUE	TRUE
40	Neuroticism	SCAD	rs4977844	G	C	-5.27E-02	9.11E-03	7.17E-09	1.25E-05	3.35E+01	TRUE	TRUE
41	Neuroticism	SCAD	rs56116032	G	A	-5.85E-02	1.05E-02	2.33E-08	1.17E-05	3.12E+01	TRUE	TRUE
42	Neuroticism	SCAD	rs56226325	T	C	-6.94E-02	1.21E-02	8.88E-09	1.24E-05	3.31E+01	TRUE	FALSE
43	Neuroticism	SCAD	rs57838764	C	T	8.06E-02	1.37E-02	3.53E-09	1.31E-05	3.49E+01	TRUE	TRUE
44	Neuroticism	SCAD	rs59970005	T	C	-6.46E-02	1.08E-02	2.20E-09	1.34E-05	3.58E+01	TRUE	TRUE
45	Neuroticism	SCAD	rs62062288	A	G	9.91E-02	1.06E-02	7.10E-21	3.29E-05	8.79E+01	TRUE	TRUE
46	Neuroticism	SCAD	rs6743916	A	G	-5.40E-02	9.58E-03	1.71E-08	1.19E-05	3.18E+01	TRUE	TRUE
47	Neuroticism	SCAD	rs6916891	T	G	7.72E-02	1.35E-02	1.09E-08	1.22E-05	3.27E+01	TRUE	FALSE
48	Neuroticism	SCAD	rs6976111	A	C	5.75E-02	9.55E-03	1.73E-09	1.36E-05	3.63E+01	TRUE	TRUE
49	Neuroticism	SCAD	rs7107293	A	G	-7.08E-02	8.77E-03	6.91E-16	2.44E-05	6.52E+01	TRUE	TRUE
50	Neuroticism	SCAD	rs7107356	G	A	6.05E-02	8.69E-03	3.50E-12	1.81E-05	4.84E+01	FALSE	FALSE
51	Neuroticism	SCAD	rs716508	T	C	-5.19E-02	9.43E-03	3.72E-08	1.13E-05	3.03E+01	TRUE	TRUE
52	Neuroticism	SCAD	rs7338774	G	A	5.77E-02	9.23E-03	4.03E-10	1.46E-05	3.91E+01	TRUE	TRUE
53	Neuroticism	SCAD	rs7502590	G	A	-7.61E-02	1.22E-02	4.33E-10	1.46E-05	3.90E+01	TRUE	TRUE
54	Neuroticism	SCAD	rs7567451	T	G	5.55E-02	9.83E-03	1.62E-08	1.20E-05	3.19E+01	TRUE	TRUE
55	Neuroticism	SCAD	rs7696796	A	G	6.01E-02	1.01E-02	2.41E-09	1.33E-05	3.56E+01	TRUE	TRUE
56	Neuroticism	SCAD	rs7869969	G	A	-5.51E-02	9.23E-03	2.40E-09	1.33E-05	3.56E+01	TRUE	TRUE
57	Neuroticism	SCAD	rs7871494	T	C	-5.52E-02	9.68E-03	1.23E-08	1.22E-05	3.24E+01	TRUE	TRUE
58	Neuroticism	SCAD	rs8053004	T	C	-5.34E-02	9.53E-03	2.08E-08	1.18E-05	3.14E+01	TRUE	TRUE
59	Neuroticism	SCAD	rs8062719	G	A	5.05E-02	9.08E-03	2.56E-08	1.16E-05	3.10E+01	TRUE	FALSE
60	Neuroticism	SCAD	rs836927	A	C	4.87E-02	8.86E-03	3.89E-08	1.13E-05	3.02E+01	TRUE	TRUE
61	Neuroticism	SCAD	rs9298995	A	G	-5.29E-02	8.88E-03	2.54E-09	1.33E-05	3.55E+01	FALSE	TRUE

62	Neuroticism	SCAD	rs9424100	G	A	-5.44E-02	9.89E-03	3.80E-08	1.13E-05	3.03E+01	TRUE	TRUE
63	Neuroticism	SCAD	rs9462364	G	A	5.21E-02	8.71E-03	2.28E-09	1.34E-05	3.57E+01	TRUE	TRUE
64	Extraversion	SCAD	rs10738513	T	G	2.32E-02	5.60E-03	3.47E-05	2.72E-04	1.72E+01	TRUE	TRUE
65	Extraversion	SCAD	rs10793451	T	C	-2.50E-02	5.90E-03	2.01E-05	2.85E-04	1.80E+01	TRUE	TRUE
66	Extraversion	SCAD	rs10891937	A	G	-2.46E-02	5.50E-03	6.94E-06	3.17E-04	2.00E+01	TRUE	TRUE
67	Extraversion	SCAD	rs10942900	A	G	2.81E-02	6.70E-03	2.64E-05	2.79E-04	1.76E+01	TRUE	TRUE
68	Extraversion	SCAD	rs111538906	T	C	-3.26E-02	8.00E-03	4.89E-05	2.63E-04	1.66E+01	TRUE	TRUE
69	Extraversion	SCAD	rs111594282	T	C	6.31E-02	1.43E-02	1.03E-05	3.09E-04	1.95E+01	TRUE	TRUE
70	Extraversion	SCAD	rs115007121	A	G	1.41E-01	3.42E-02	3.92E-05	2.69E-04	1.69E+01	TRUE	TRUE
71	Extraversion	SCAD	rs11645166	T	C	3.57E-02	8.30E-03	1.56E-05	2.93E-04	1.85E+01	TRUE	TRUE
72	Extraversion	SCAD	rs12109897	A	G	-4.90E-02	1.05E-02	2.97E-06	3.45E-04	2.18E+01	TRUE	TRUE
73	Extraversion	SCAD	rs13012149	A	G	2.38E-02	5.50E-03	1.66E-05	2.97E-04	1.87E+01	TRUE	TRUE
74	Extraversion	SCAD	rs13410278	A	G	-6.85E-02	1.49E-02	4.38E-06	3.35E-04	2.11E+01	TRUE	TRUE
75	Extraversion	SCAD	rs1362413	A	G	2.29E-02	5.40E-03	2.47E-05	2.85E-04	1.80E+01	TRUE	TRUE
76	Extraversion	SCAD	rs1382462	A	G	3.44E-02	8.30E-03	3.65E-05	2.72E-04	1.72E+01	TRUE	TRUE
77	Extraversion	SCAD	rs1463436	A	G	-2.52E-02	5.80E-03	1.15E-05	2.99E-04	1.89E+01	TRUE	TRUE
78	Extraversion	SCAD	rs146794654	T	C	7.91E-02	1.91E-02	3.57E-05	2.72E-04	1.72E+01	TRUE	TRUE
79	Extraversion	SCAD	rs1666057	A	G	-2.72E-02	6.20E-03	1.01E-05	3.05E-04	1.92E+01	FALSE	TRUE
80	Extraversion	SCAD	rs170135	A	G	3.14E-02	7.40E-03	2.31E-05	2.86E-04	1.80E+01	TRUE	TRUE
81	Extraversion	SCAD	rs17025910	T	G	-1.53E-01	3.53E-02	1.51E-05	2.97E-04	1.87E+01	TRUE	TRUE
82	Extraversion	SCAD	rs17237193	A	G	-4.85E-02	1.12E-02	1.55E-05	2.97E-04	1.88E+01	TRUE	TRUE
83	Extraversion	SCAD	rs179254	T	C	2.37E-02	5.60E-03	2.03E-05	2.84E-04	1.79E+01	TRUE	TRUE
84	Extraversion	SCAD	rs2024488	A	G	-3.03E-02	5.90E-03	2.94E-07	4.18E-04	2.64E+01	TRUE	TRUE
85	Extraversion	SCAD	rs2040191	A	G	-3.88E-02	9.00E-03	1.71E-05	2.95E-04	1.86E+01	FALSE	TRUE
86	Extraversion	SCAD	rs2164273	A	G	-2.44E-02	6.00E-03	4.08E-05	2.62E-04	1.65E+01	TRUE	FALSE

87	Extraversion	SCAD	rs2244544	T	C	-2.21E-02	5.40E-03	4.02E-05	2.66E-04	1.67E+01	TRUE	TRUE
88	Extraversion	SCAD	rs2322815	T	C	-2.63E-02	6.10E-03	1.45E-05	2.95E-04	1.86E+01	TRUE	TRUE
89	Extraversion	SCAD	rs2332941	A	C	2.35E-02	5.50E-03	2.05E-05	2.90E-04	1.83E+01	TRUE	TRUE
90	Extraversion	SCAD	rs2965086	T	C	-2.45E-02	6.00E-03	4.86E-05	2.64E-04	1.67E+01	FALSE	TRUE
91	Extraversion	SCAD	rs303839	T	G	-3.70E-02	8.50E-03	1.40E-05	3.01E-04	1.89E+01	TRUE	TRUE
92	Extraversion	SCAD	rs34041659	T	C	3.21E-02	7.40E-03	1.52E-05	2.98E-04	1.88E+01	TRUE	TRUE
93	Extraversion	SCAD	rs34811382	T	C	7.73E-02	1.77E-02	1.25E-05	3.03E-04	1.91E+01	TRUE	TRUE
94	Extraversion	SCAD	rs35429502	T	C	2.46E-02	6.00E-03	4.41E-05	2.67E-04	1.68E+01	TRUE	TRUE
95	Extraversion	SCAD	rs3849571	A	C	-2.53E-02	5.70E-03	7.92E-06	3.12E-04	1.97E+01	TRUE	TRUE
96	Extraversion	SCAD	rs42385	A	C	-2.66E-02	5.90E-03	6.92E-06	3.22E-04	2.03E+01	TRUE	TRUE
97	Extraversion	SCAD	rs4810656	A	G	-2.24E-02	5.50E-03	4.84E-05	2.63E-04	1.66E+01	TRUE	TRUE
98	Extraversion	SCAD	rs57959293	T	C	7.68E-02	1.78E-02	1.63E-05	2.95E-04	1.86E+01	TRUE	TRUE
99	Extraversion	SCAD	rs62357223	A	G	-2.38E-02	5.50E-03	1.31E-05	2.97E-04	1.87E+01	TRUE	TRUE
100	Extraversion	SCAD	rs6442336	T	C	3.04E-02	6.60E-03	4.84E-06	3.36E-04	2.12E+01	FALSE	TRUE
101	Extraversion	SCAD	rs6487099	A	G	2.54E-02	6.20E-03	4.79E-05	2.66E-04	1.68E+01	TRUE	TRUE
102	Extraversion	SCAD	rs668013	A	G	-3.65E-02	8.20E-03	8.68E-06	3.14E-04	1.98E+01	TRUE	TRUE
103	Extraversion	SCAD	rs67962081	T	G	-2.84E-02	7.00E-03	4.77E-05	2.61E-04	1.65E+01	TRUE	TRUE
104	Extraversion	SCAD	rs699946	A	G	3.01E-02	7.40E-03	4.50E-05	2.62E-04	1.65E+01	TRUE	TRUE
105	Extraversion	SCAD	rs7002652	A	C	-2.50E-02	5.40E-03	4.09E-06	3.40E-04	2.14E+01	TRUE	TRUE
106	Extraversion	SCAD	rs71245954	A	G	-5.40E-02	1.32E-02	4.08E-05	2.65E-04	1.67E+01	TRUE	TRUE
107	Extraversion	SCAD	rs7244637	A	C	-3.33E-02	7.80E-03	2.12E-05	2.89E-04	1.82E+01	TRUE	TRUE
108	Extraversion	SCAD	rs75317441	T	C	-1.28E-01	3.12E-02	4.23E-05	2.67E-04	1.68E+01	TRUE	TRUE
109	Extraversion	SCAD	rs7605005	A	G	3.29E-02	7.60E-03	1.40E-05	2.97E-04	1.87E+01	TRUE	TRUE
110	Extraversion	SCAD	rs76669452	A	G	-4.51E-02	9.90E-03	5.65E-06	3.29E-04	2.08E+01	TRUE	TRUE
111	Extraversion	SCAD	rs7718185	T	G	2.24E-02	5.50E-03	4.58E-05	2.63E-04	1.66E+01	TRUE	TRUE

112	Extraversion	SCAD	rs79450314	A	C	8.81E-02	2.14E-02	3.86E-05	2.69E-04	1.69E+01	TRUE	TRUE
113	Extraversion	SCAD	rs797182	A	G	-2.77E-02	5.60E-03	6.67E-07	3.88E-04	2.45E+01	TRUE	TRUE
114	Extraversion	SCAD	rs79870297	T	C	-3.23E-02	6.60E-03	9.92E-07	3.80E-04	2.39E+01	TRUE	TRUE
115	Extraversion	SCAD	rs8010306	A	G	6.29E-02	1.28E-02	8.73E-07	3.83E-04	2.41E+01	TRUE	TRUE
116	Extraversion	SCAD	rs932570	T	C	-2.82E-02	6.00E-03	2.71E-06	3.50E-04	2.21E+01	TRUE	TRUE
117	Extraversion	SCAD	rs9510614	T	C	-4.79E-02	1.18E-02	4.82E-05	2.61E-04	1.65E+01	TRUE	TRUE
118	Openness	SCAD	rs10812072	C	T	2.94E-01	7.13E-02	3.64E-05	9.80E-04	1.70E+01	TRUE	TRUE
119	Openness	SCAD	rs10829186	G	A	-4.15E-01	8.74E-02	2.13E-06	1.29E-03	2.25E+01	TRUE	TRUE
120	Openness	SCAD	rs10932966	A	C	5.25E-01	1.04E-01	4.80E-07	1.46E-03	2.54E+01	TRUE	TRUE
121	Openness	SCAD	rs11157383	G	A	-3.06E-01	7.48E-02	4.28E-05	9.62E-04	1.67E+01	TRUE	TRUE
122	Openness	SCAD	rs11628275	A	G	-8.42E-01	1.97E-01	1.96E-05	1.05E-03	1.82E+01	TRUE	TRUE
123	Openness	SCAD	rs11685318	A	C	-3.84E-01	8.70E-02	1.02E-05	1.12E-03	1.95E+01	TRUE	TRUE
124	Openness	SCAD	rs11786021	C	T	-4.06E-01	8.97E-02	6.01E-06	1.18E-03	2.05E+01	TRUE	FALSE
125	Openness	SCAD	rs12240003	C	T	-3.57E-01	8.78E-02	4.82E-05	9.50E-04	1.65E+01	TRUE	TRUE
126	Openness	SCAD	rs12451097	A	G	9.16E-01	2.16E-01	2.24E-05	1.03E-03	1.80E+01	TRUE	TRUE
127	Openness	SCAD	rs12815688	T	C	5.98E-01	1.31E-01	4.91E-06	1.20E-03	2.09E+01	TRUE	TRUE
128	Openness	SCAD	rs13179075	C	T	-5.06E-01	1.08E-01	2.59E-06	1.27E-03	2.21E+01	TRUE	TRUE
129	Openness	SCAD	rs1477268	C	T	4.80E-01	8.65E-02	2.79E-08	1.77E-03	3.08E+01	TRUE	TRUE
130	Openness	SCAD	rs16970755	A	G	-3.74E-01	7.92E-02	2.33E-06	1.28E-03	2.23E+01	FALSE	TRUE
131	Openness	SCAD	rs16984966	C	T	3.80E-01	8.83E-02	1.66E-05	1.07E-03	1.85E+01	TRUE	TRUE
132	Openness	SCAD	rs17066856	C	T	-5.26E-01	1.20E-01	1.22E-05	1.10E-03	1.91E+01	TRUE	TRUE
133	Openness	SCAD	rs213786	G	A	3.80E-01	9.21E-02	3.69E-05	9.79E-04	1.70E+01	TRUE	TRUE
134	Openness	SCAD	rs2230491	T	C	4.52E-01	1.01E-01	7.06E-06	1.16E-03	2.02E+01	TRUE	TRUE
135	Openness	SCAD	rs2980896	C	T	-4.30E-01	1.02E-01	2.42E-05	1.02E-03	1.78E+01	TRUE	TRUE
136	Openness	SCAD	rs3859314	G	A	-4.15E-01	1.01E-01	3.97E-05	9.70E-04	1.69E+01	TRUE	TRUE

137	Openness	SCAD	rs4246037	T	C	3.11E-01	7.35E-02	2.36E-05	1.03E-03	1.79E+01	TRUE	TRUE
138	Openness	SCAD	rs4334580	A	G	-3.19E-01	7.28E-02	1.18E-05	1.11E-03	1.92E+01	TRUE	TRUE
139	Openness	SCAD	rs4515089	C	A	5.82E-01	1.31E-01	8.38E-06	1.14E-03	1.98E+01	TRUE	TRUE
140	Openness	SCAD	rs4861306	T	C	2.88E-01	7.07E-02	4.54E-05	9.56E-04	1.66E+01	TRUE	TRUE
141	Openness	SCAD	rs6489657	A	G	3.44E-01	7.95E-02	1.54E-05	1.08E-03	1.87E+01	TRUE	TRUE
142	Openness	SCAD	rs677035	T	C	-5.59E-01	1.19E-01	2.61E-06	1.27E-03	2.21E+01	TRUE	TRUE
143	Openness	SCAD	rs7003960	G	A	3.04E-01	7.22E-02	2.63E-05	1.02E-03	1.77E+01	TRUE	TRUE
144	Openness	SCAD	rs7827536	G	T	-5.94E-01	1.31E-01	6.13E-06	1.18E-03	2.05E+01	FALSE	TRUE
145	Openness	SCAD	rs7997606	G	A	3.50E-01	8.06E-02	1.37E-05	1.09E-03	1.89E+01	TRUE	TRUE
146	Openness	SCAD	rs885127	A	G	-3.21E-01	7.67E-02	2.83E-05	1.01E-03	1.75E+01	FALSE	TRUE
147	Openness	SCAD	rs947473	C	A	4.01E-01	8.76E-02	4.75E-06	1.20E-03	2.09E+01	TRUE	TRUE
148	Agreeableness	SCAD	rs1060618	G	A	3.79E-01	8.72E-02	1.35E-05	1.09E-03	1.89E+01	TRUE	TRUE
149	Agreeableness	SCAD	rs10880936	C	T	-5.77E-01	1.39E-01	3.40E-05	9.88E-04	1.72E+01	TRUE	TRUE
150	Agreeableness	SCAD	rs11086214	C	T	3.89E-01	9.25E-02	2.68E-05	1.01E-03	1.76E+01	TRUE	TRUE
151	Agreeableness	SCAD	rs11174704	C	T	-2.58E-01	6.27E-02	3.97E-05	9.71E-04	1.69E+01	TRUE	TRUE
152	Agreeableness	SCAD	rs11588414	T	C	5.31E-01	1.22E-01	1.31E-05	1.09E-03	1.90E+01	TRUE	TRUE
153	Agreeableness	SCAD	rs12149802	T	C	4.24E-01	9.78E-02	1.46E-05	1.08E-03	1.88E+01	TRUE	TRUE
154	Agreeableness	SCAD	rs12813504	G	A	2.83E-01	6.59E-02	1.74E-05	1.06E-03	1.84E+01	TRUE	TRUE
155	Agreeableness	SCAD	rs12920571	G	A	-5.65E-01	1.39E-01	4.54E-05	9.57E-04	1.66E+01	TRUE	TRUE
156	Agreeableness	SCAD	rs12945271	T	C	2.94E-01	6.36E-02	3.93E-06	1.23E-03	2.13E+01	TRUE	TRUE
157	Agreeableness	SCAD	rs13113475	A	G	2.79E-01	6.86E-02	4.78E-05	9.52E-04	1.66E+01	TRUE	TRUE
158	Agreeableness	SCAD	rs17619532	G	A	-3.78E-01	9.13E-02	3.50E-05	9.85E-04	1.71E+01	TRUE	TRUE
159	Agreeableness	SCAD	rs1794590	C	T	-3.10E-01	7.07E-02	1.19E-05	1.10E-03	1.92E+01	TRUE	TRUE
160	Agreeableness	SCAD	rs2174190	A	C	-4.29E-01	1.05E-01	4.48E-05	9.58E-04	1.67E+01	TRUE	TRUE
161	Agreeableness	SCAD	rs2486053	C	T	3.07E-01	7.08E-02	1.45E-05	1.08E-03	1.88E+01	TRUE	TRUE

162	Agreeableness	SCAD	rs4468514	C	T	2.72E-01	6.27E-02	1.38E-05	1.09E-03	1.89E+01	TRUE	TRUE
163	Agreeableness	SCAD	rs4679142	T	C	2.54E-01	6.17E-02	3.95E-05	9.71E-04	1.69E+01	TRUE	TRUE
164	Agreeableness	SCAD	rs4817527	A	G	-2.80E-01	6.25E-02	7.70E-06	1.15E-03	2.00E+01	TRUE	TRUE
165	Agreeableness	SCAD	rs4975235	T	G	3.38E-01	8.07E-02	2.77E-05	1.01E-03	1.76E+01	TRUE	TRUE
166	Agreeableness	SCAD	rs6137005	G	A	-2.65E-01	6.21E-02	1.94E-05	1.05E-03	1.82E+01	TRUE	TRUE
167	Agreeableness	SCAD	rs705066	G	A	-2.65E-01	6.48E-02	4.40E-05	9.61E-04	1.67E+01	TRUE	TRUE
168	Agreeableness	SCAD	rs7100191	C	T	2.58E-01	6.17E-02	2.96E-05	1.00E-03	1.74E+01	TRUE	TRUE
169	Agreeableness	SCAD	rs7108752	G	A	-3.30E-01	7.37E-02	7.58E-06	1.15E-03	2.00E+01	TRUE	TRUE
170	Agreeableness	SCAD	rs7405603	T	C	-3.74E-01	8.81E-02	2.23E-05	1.04E-03	1.80E+01	TRUE	TRUE
171	Agreeableness	SCAD	rs765058	G	A	-3.03E-01	6.89E-02	1.09E-05	1.11E-03	1.94E+01	TRUE	TRUE
172	Agreeableness	SCAD	rs8029033	T	C	3.58E-01	7.37E-02	1.23E-06	1.35E-03	2.36E+01	TRUE	TRUE
173	Agreeableness	SCAD	rs8037245	C	T	-3.35E-01	7.91E-02	2.33E-05	1.03E-03	1.79E+01	TRUE	TRUE
174	Agreeableness	SCAD	rs9668437	C	T	-4.19E-01	9.88E-02	2.24E-05	1.03E-03	1.80E+01	TRUE	TRUE
175	Agreeableness	SCAD	rs967079	C	T	2.75E-01	6.24E-02	1.03E-05	1.12E-03	1.94E+01	TRUE	TRUE
176	Agreeableness	SCAD	rs9838033	G	T	-2.68E-01	6.28E-02	1.94E-05	1.05E-03	1.82E+01	TRUE	TRUE
177	Conscientiousness	SCAD	rs10178585	A	G	-3.49E-01	7.75E-02	6.61E-06	1.17E-03	2.03E+01	TRUE	TRUE
178	Conscientiousness	SCAD	rs10754317	A	G	-3.58E-01	8.51E-02	2.60E-05	1.02E-03	1.77E+01	TRUE	TRUE
179	Conscientiousness	SCAD	rs10834157	T	C	3.28E-01	8.03E-02	4.44E-05	9.59E-04	1.67E+01	TRUE	TRUE
180	Conscientiousness	SCAD	rs11057538	G	A	-5.67E-01	1.29E-01	1.06E-05	1.12E-03	1.94E+01	TRUE	TRUE
181	Conscientiousness	SCAD	rs11764509	C	T	-3.15E-01	7.60E-02	3.43E-05	9.88E-04	1.72E+01	TRUE	TRUE
182	Conscientiousness	SCAD	rs12541726	A	G	-4.97E-01	1.21E-01	4.12E-05	9.66E-04	1.68E+01	TRUE	TRUE
183	Conscientiousness	SCAD	rs12933226	G	A	3.19E-01	7.75E-02	3.81E-05	9.74E-04	1.69E+01	TRUE	TRUE
184	Conscientiousness	SCAD	rs1539569	C	T	-3.69E-01	8.95E-02	3.71E-05	9.78E-04	1.70E+01	TRUE	TRUE
185	Conscientiousness	SCAD	rs17311358	A	G	-8.44E-01	1.85E-01	4.78E-06	1.20E-03	2.09E+01	TRUE	TRUE
186	Conscientiousness	SCAD	rs2044279	G	T	-5.19E-01	1.28E-01	4.85E-05	9.49E-04	1.65E+01	TRUE	TRUE

187	Conscientiousness	SCAD	rs207420	C	T	-7.45E-01	1.67E-01	7.97E-06	1.15E-03	1.99E+01	TRUE	TRUE
188	Conscientiousness	SCAD	rs2234570	T	C	-5.30E-01	1.27E-01	2.96E-05	1.00E-03	1.74E+01	TRUE	TRUE
189	Conscientiousness	SCAD	rs2270412	A	G	4.48E-01	1.01E-01	8.29E-06	1.14E-03	1.99E+01	TRUE	TRUE
190	Conscientiousness	SCAD	rs2330993	G	A	-3.47E-01	8.20E-02	2.40E-05	1.03E-03	1.79E+01	TRUE	TRUE
191	Conscientiousness	SCAD	rs233826	C	T	3.86E-01	8.23E-02	2.70E-06	1.27E-03	2.20E+01	TRUE	TRUE
192	Conscientiousness	SCAD	rs2356798	T	G	3.47E-01	7.52E-02	3.87E-06	1.23E-03	2.13E+01	TRUE	TRUE
193	Conscientiousness	SCAD	rs2576037	T	C	-4.08E-01	7.47E-02	4.91E-08	1.71E-03	2.98E+01	TRUE	TRUE
194	Conscientiousness	SCAD	rs2881301	T	G	3.43E-01	7.75E-02	9.38E-06	1.13E-03	1.96E+01	TRUE	TRUE
195	Conscientiousness	SCAD	rs4750290	G	A	6.15E-01	1.43E-01	1.76E-05	1.06E-03	1.84E+01	TRUE	TRUE
196	Conscientiousness	SCAD	rs4780150	T	C	6.28E-01	1.47E-01	1.98E-05	1.05E-03	1.82E+01	TRUE	TRUE
197	Conscientiousness	SCAD	rs569833	C	T	3.62E-01	8.47E-02	1.97E-05	1.05E-03	1.82E+01	TRUE	TRUE
198	Conscientiousness	SCAD	rs6926133	C	A	-3.81E-01	9.33E-02	4.51E-05	9.57E-04	1.66E+01	TRUE	TRUE
199	Conscientiousness	SCAD	rs7029990	C	A	-4.51E-01	1.05E-01	1.78E-05	1.06E-03	1.84E+01	TRUE	TRUE
200	Conscientiousness	SCAD	rs7252142	G	A	-3.31E-01	7.77E-02	2.05E-05	1.04E-03	1.81E+01	TRUE	TRUE
201	Conscientiousness	SCAD	rs7634557	C	T	4.38E-01	1.04E-01	2.49E-05	1.02E-03	1.78E+01	TRUE	TRUE
202	Conscientiousness	SCAD	rs8087497	A	G	-6.68E-01	1.61E-01	3.36E-05	9.90E-04	1.72E+01	TRUE	TRUE
203	Conscientiousness	SCAD	rs9354878	G	A	-3.79E-01	9.16E-02	3.48E-05	9.85E-04	1.71E+01	TRUE	TRUE
204	Conscientiousness	SCAD	rs9364416	G	A	-3.97E-01	9.40E-02	2.45E-05	1.02E-03	1.78E+01	TRUE	TRUE
205	Conscientiousness	SCAD	rs9710693	C	T	3.68E-01	8.97E-02	4.12E-05	9.67E-04	1.68E+01	TRUE	TRUE
206	Conscientiousness	SCAD	rs9844675	A	C	7.57E-01	1.86E-01	4.80E-05	9.50E-04	1.65E+01	TRUE	TRUE
207	SCAD	Neuroticism	rs10826820	A	C	2.93E-01	5.29E-02	3.26E-08	2.72E-03	3.06E+01	TRUE	TRUE
208	SCAD	Neuroticism	rs10851839	A	T	2.81E-01	4.29E-02	5.51E-11	3.82E-03	4.30E+01	TRUE	TRUE
209	SCAD	Neuroticism	rs11021221	A	T	3.83E-01	4.88E-02	4.11E-15	5.46E-03	6.15E+01	TRUE	TRUE
210	SCAD	Neuroticism	rs11172113	C	T	-4.82E-01	4.18E-02	9.03E-31	1.17E-02	1.33E+02	FALSE	TRUE
211	SCAD	Neuroticism	rs1146473	C	T	2.76E-01	4.74E-02	5.82E-09	3.01E-03	3.39E+01	TRUE	TRUE

212	SCAD	Neuroticism	rs11838776	A	G	-4.07E-01	4.66E-02	2.46E-18	6.76E-03	7.63E+01	TRUE	TRUE
213	SCAD	Neuroticism	rs137507	C	T	-3.21E-01	5.82E-02	3.30E-08	2.71E-03	3.05E+01	TRUE	TRUE
214	SCAD	Neuroticism	rs1507928	C	T	2.25E-01	3.92E-02	8.94E-09	2.94E-03	3.30E+01	TRUE	TRUE
215	SCAD	Neuroticism	rs1689040	T	C	-2.47E-01	4.01E-02	7.04E-10	3.38E-03	3.81E+01	TRUE	TRUE
216	SCAD	Neuroticism	rs2325944	A	C	-2.58E-01	4.65E-02	2.87E-08	2.74E-03	3.08E+01	TRUE	TRUE
217	SCAD	Neuroticism	rs2736923	A	G	3.65E-01	6.67E-02	4.58E-08	2.66E-03	2.99E+01	TRUE	TRUE
218	SCAD	Neuroticism	rs28451064	A	G	-7.12E-01	7.27E-02	1.16E-22	8.49E-03	9.59E+01	TRUE	TRUE
219	SCAD	Neuroticism	rs28627182	G	T	5.64E-01	9.98E-02	1.62E-08	2.84E-03	3.19E+01	TRUE	TRUE
220	SCAD	Neuroticism	rs34370185	T	G	2.95E-01	4.16E-02	1.42E-12	4.46E-03	5.02E+01	TRUE	TRUE
221	SCAD	Neuroticism	rs35982037	C	T	6.91E-01	1.21E-01	1.08E-08	2.91E-03	3.27E+01	TRUE	TRUE
222	SCAD	Neuroticism	rs4947307	G	A	7.28E-01	1.05E-01	3.21E-12	4.32E-03	4.86E+01	FALSE	TRUE
223	SCAD	Neuroticism	rs4970935	T	C	-5.43E-01	4.16E-02	6.14E-39	1.50E-02	1.70E+02	FALSE	TRUE
224	SCAD	Neuroticism	rs57871297	T	C	8.03E-01	1.35E-01	2.98E-09	3.13E-03	3.52E+01	TRUE	TRUE
225	SCAD	Neuroticism	rs58638188	G	T	5.04E-01	8.12E-02	5.24E-10	3.43E-03	3.85E+01	TRUE	TRUE
226	SCAD	Neuroticism	rs6023105	T	C	9.65E-01	1.72E-01	2.02E-08	2.80E-03	3.15E+01	TRUE	TRUE
227	SCAD	Neuroticism	rs6828005	A	G	-2.57E-01	3.86E-02	2.60E-11	3.95E-03	4.44E+01	TRUE	TRUE
228	SCAD	Neuroticism	rs71371152	A	C	8.91E-01	1.55E-01	9.74E-09	2.93E-03	3.29E+01	FALSE	TRUE
229	SCAD	Neuroticism	rs7174973	G	A	4.30E-01	5.83E-02	1.60E-13	4.83E-03	5.44E+01	TRUE	TRUE
230	SCAD	Neuroticism	rs73102285	G	A	2.42E-01	4.23E-02	1.05E-08	2.91E-03	3.27E+01	TRUE	TRUE
231	SCAD	Neuroticism	rs7385111	A	C	-3.04E-01	4.34E-02	2.45E-12	4.35E-03	4.90E+01	TRUE	TRUE
232	SCAD	Neuroticism	rs9349379	G	A	-4.95E-01	4.19E-02	2.88E-32	1.23E-02	1.40E+02	TRUE	TRUE
233	SCAD	Neuroticism	rs9947850	A	G	-4.41E-01	7.22E-02	9.61E-10	3.32E-03	3.74E+01	FALSE	TRUE
234	SCAD	Extraversion	rs10826820	A	C	2.93E-01	5.29E-02	3.26E-08	2.72E-03	3.06E+01	TRUE	TRUE
235	SCAD	Extraversion	rs11172113	C	T	-4.82E-01	4.18E-02	9.03E-31	1.17E-02	1.33E+02	TRUE	TRUE
236	SCAD	Extraversion	rs1146473	C	T	2.76E-01	4.74E-02	5.82E-09	3.01E-03	3.39E+01	FALSE	TRUE

237	SCAD	Extraversion	rs11838776	A	G	-4.07E-01	4.66E-02	2.46E-18	6.76E-03	7.63E+01	TRUE	TRUE
238	SCAD	Extraversion	rs137507	C	T	-3.21E-01	5.82E-02	3.30E-08	2.71E-03	3.05E+01	TRUE	TRUE
239	SCAD	Extraversion	rs1507928	C	T	2.25E-01	3.92E-02	8.94E-09	2.94E-03	3.30E+01	TRUE	TRUE
240	SCAD	Extraversion	rs1689040	T	C	-2.47E-01	4.01E-02	7.04E-10	3.38E-03	3.81E+01	TRUE	TRUE
241	SCAD	Extraversion	rs2325944	A	C	-2.58E-01	4.65E-02	2.87E-08	2.74E-03	3.08E+01	TRUE	TRUE
242	SCAD	Extraversion	rs2736923	A	G	3.65E-01	6.67E-02	4.58E-08	2.66E-03	2.99E+01	TRUE	TRUE
243	SCAD	Extraversion	rs28451064	A	G	-7.12E-01	7.27E-02	1.16E-22	8.49E-03	9.59E+01	TRUE	TRUE
244	SCAD	Extraversion	rs28627182	G	T	5.64E-01	9.98E-02	1.62E-08	2.84E-03	3.19E+01	TRUE	TRUE
245	SCAD	Extraversion	rs34370185	T	G	2.95E-01	4.16E-02	1.42E-12	4.46E-03	5.02E+01	TRUE	TRUE
246	SCAD	Extraversion	rs57871297	T	C	8.03E-01	1.35E-01	2.98E-09	3.13E-03	3.52E+01	TRUE	TRUE
247	SCAD	Extraversion	rs58638188	G	T	5.04E-01	8.12E-02	5.24E-10	3.43E-03	3.85E+01	TRUE	TRUE
248	SCAD	Extraversion	rs6023105	T	C	9.65E-01	1.72E-01	2.02E-08	2.80E-03	3.15E+01	TRUE	TRUE
249	SCAD	Extraversion	rs6828005	A	G	-2.57E-01	3.86E-02	2.60E-11	3.95E-03	4.44E+01	TRUE	TRUE
250	SCAD	Extraversion	rs7174973	G	A	4.30E-01	5.83E-02	1.60E-13	4.83E-03	5.44E+01	TRUE	TRUE
251	SCAD	Extraversion	rs73102285	G	A	2.42E-01	4.23E-02	1.05E-08	2.91E-03	3.27E+01	TRUE	TRUE
252	SCAD	Extraversion	rs7385111	A	C	-3.04E-01	4.34E-02	2.45E-12	4.35E-03	4.90E+01	TRUE	TRUE
253	SCAD	Extraversion	rs9349379	G	A	-4.95E-01	4.19E-02	2.88E-32	1.23E-02	1.40E+02	TRUE	TRUE
254	SCAD	Extraversion	rs9947850	A	G	-4.41E-01	7.22E-02	9.61E-10	3.32E-03	3.74E+01	TRUE	TRUE
255	SCAD	Openness	rs11172113	C	T	-4.82E-01	4.18E-02	9.03E-31	1.17E-02	1.33E+02	TRUE	TRUE
256	SCAD	Openness	rs1146473	C	T	2.76E-01	4.74E-02	5.82E-09	3.01E-03	3.39E+01	TRUE	TRUE
257	SCAD	Openness	rs137507	C	T	-3.21E-01	5.82E-02	3.30E-08	2.71E-03	3.05E+01	FALSE	TRUE
258	SCAD	Openness	rs1507928	C	T	2.25E-01	3.92E-02	8.94E-09	2.94E-03	3.30E+01	TRUE	TRUE
259	SCAD	Openness	rs2325944	A	C	-2.58E-01	4.65E-02	2.87E-08	2.74E-03	3.08E+01	TRUE	TRUE
260	SCAD	Openness	rs2736923	A	G	3.65E-01	6.67E-02	4.58E-08	2.66E-03	2.99E+01	TRUE	TRUE
261	SCAD	Openness	rs6828005	A	G	-2.57E-01	3.86E-02	2.60E-11	3.95E-03	4.44E+01	TRUE	TRUE

262	SCAD	Openness	rs7174973	G	A	4.30E-01	5.83E-02	1.60E-13	4.83E-03	5.44E+01	TRUE	TRUE
263	SCAD	Openness	rs9349379	G	A	-4.95E-01	4.19E-02	2.88E-32	1.23E-02	1.40E+02	TRUE	TRUE
264	SCAD	Openness	rs9947850	A	G	-4.41E-01	7.22E-02	9.61E-10	3.32E-03	3.74E+01	TRUE	TRUE
265	SCAD	Agreeableness	rs11172113	C	T	-4.82E-01	4.18E-02	9.03E-31	1.17E-02	1.33E+02	TRUE	TRUE
266	SCAD	Agreeableness	rs1146473	C	T	2.76E-01	4.74E-02	5.82E-09	3.01E-03	3.39E+01	TRUE	TRUE
267	SCAD	Agreeableness	rs137507	C	T	-3.21E-01	5.82E-02	3.30E-08	2.71E-03	3.05E+01	TRUE	TRUE
268	SCAD	Agreeableness	rs1507928	C	T	2.25E-01	3.92E-02	8.94E-09	2.94E-03	3.30E+01	TRUE	TRUE
269	SCAD	Agreeableness	rs2325944	A	C	-2.58E-01	4.65E-02	2.87E-08	2.74E-03	3.08E+01	TRUE	TRUE
270	SCAD	Agreeableness	rs2736923	A	G	3.65E-01	6.67E-02	4.58E-08	2.66E-03	2.99E+01	TRUE	TRUE
271	SCAD	Agreeableness	rs6828005	A	G	-2.57E-01	3.86E-02	2.60E-11	3.95E-03	4.44E+01	TRUE	TRUE
272	SCAD	Agreeableness	rs7174973	G	A	4.30E-01	5.83E-02	1.60E-13	4.83E-03	5.44E+01	TRUE	TRUE
273	SCAD	Agreeableness	rs9349379	G	A	-4.95E-01	4.19E-02	2.88E-32	1.23E-02	1.40E+02	TRUE	TRUE
274	SCAD	Agreeableness	rs9947850	A	G	-4.41E-01	7.22E-02	9.61E-10	3.32E-03	3.74E+01	TRUE	TRUE
275	SCAD	Conscientiousness	rs11172113	C	T	-4.82E-01	4.18E-02	9.03E-31	1.17E-02	1.33E+02	TRUE	TRUE
276	SCAD	Conscientiousness	rs1146473	C	T	2.76E-01	4.74E-02	5.82E-09	3.01E-03	3.39E+01	TRUE	TRUE
277	SCAD	Conscientiousness	rs137507	C	T	-3.21E-01	5.82E-02	3.30E-08	2.71E-03	3.05E+01	TRUE	TRUE
278	SCAD	Conscientiousness	rs1507928	C	T	2.25E-01	3.92E-02	8.94E-09	2.94E-03	3.30E+01	TRUE	TRUE
279	SCAD	Conscientiousness	rs2325944	A	C	-2.58E-01	4.65E-02	2.87E-08	2.74E-03	3.08E+01	TRUE	TRUE
280	SCAD	Conscientiousness	rs2736923	A	G	3.65E-01	6.67E-02	4.58E-08	2.66E-03	2.99E+01	TRUE	TRUE
281	SCAD	Conscientiousness	rs6828005	A	G	-2.57E-01	3.86E-02	2.60E-11	3.95E-03	4.44E+01	TRUE	TRUE
282	SCAD	Conscientiousness	rs7174973	G	A	4.30E-01	5.83E-02	1.60E-13	4.83E-03	5.44E+01	TRUE	TRUE
283	SCAD	Conscientiousness	rs9349379	G	A	-4.95E-01	4.19E-02	2.88E-32	1.23E-02	1.40E+02	TRUE	TRUE
284	SCAD	Conscientiousness	rs9947850	A	G	-4.41E-01	7.22E-02	9.61E-10	3.32E-03	3.74E+01	TRUE	TRUE

Supplementary Table3. Heterogeneity and horizontal pleiotropy of the instrumental variables in the Reverse Mendelian randomization.

	Outcome	Heterogeneity Test		Pleiotropy test	MR-PRESSO	
		Cochran's Q Test (P value)		Egger Intercept (P value)	Distortion Test	Global Test
		IVW	MR-Egger	MR-Egger	Outliers	P value
UVMR	Neuroticism	0.84	0.81	0.64	NA	0.84
	Extraversion	0.61	0.56	0.71	NA	0.64
	Openness	1.00	0.99	0.83	NA	1.00
	Agreeableness	0.69	0.61	0.66	NA	0.75
	Conscientiousness	0.55	0.85	0.09	NA	0.59

UVMR, Univariable MR; IVW, inverse variance weighting

Supplementary Figure 2. The figure legends are required to have the same font as the main text, 12 point normal Times New Roman, single spaced. Please use a single paragraph for each legend and prepare the figures keeping in mind the PDF layout.

Supplementary Figure 3. The figure legends are required to have the same font as the main text, 12 point normal Times New Roman, single spaced. Please use a single paragraph for each legend and prepare the figures keeping in mind the PDF layout.

Supplementary Figure 4. The figure legends are required to have the same font as the main text, 12 point normal Times New Roman, single spaced. Please use a single paragraph for each legend and prepare the figures keeping in mind the PDF layout.

Supplementary Figure 5. The figure legends are required to have the same font as the main text, 12 point normal Times New Roman, single spaced. Please use a single paragraph for each legend and prepare the figures keeping in mind the PDF layout.

Supplementary Figure 6. The figure legends are required to have the same font as the main text, 12 point normal Times New Roman, single spaced. Please use a single paragraph for each legend and prepare the figures keeping in mind the PDF layout.
